# Causal evidence for dorsal stream involvement in object shape recognition: A tractography-guided ccPAS Registered Report study

**DOI:** 10.1162/IMAG.a.1345

**Published:** 2026-09-08

**Authors:** Elena Bertacco, Francesca Saviola, Edoardo Paolini, Francesca Benedetta Pizzini, Nicolò Cardobi, Agnese Tamanti, Silvia Francesca Storti, Debora Brignani, Silvia Savazzi, Daniele Corbo, Chiara Mazzi, Chiara Bagattini

**Affiliations:** Department of Neuroscience, Biomedicine and Movement Sciences, University of Verona, Verona, Italy; Neuro-X Institute, Ecole Polytechnique Fédérale de Lausanne (EPFL), Geneva, Switzerland; Department of Medical and Surgical Specialties, Radiological Sciences and Public Health, Università di Brescia, Brescia, Italy; Department of Engineering for Innovation Medicine, University of Verona, Verona, Italy; Department of Diagnostics and Public Health, University of Verona, Verona, Italy; Department of Clinical and Experimental Sciences, Università di Brescia, Brescia, Italy

**Keywords:** dorsal visual pathway, global shape, transcranial magnetic stimulation (TMS), DWI, connectivity

## Abstract

Visual experience requires orchestration of intricate brain connections. One of the most influential models of human visual information processing posits two segregated streams and postulates that conscious object recognition relies exclusively on the ventral visual stream. However, recent evidence suggests that the dorsal stream is involved in encoding crucial attributes of visual object recognition, such as global shape. The specific role of the dorsal stream is controversial in this context. Through an individualized multimodal approach, we investigated the contribution of the dorsal pathway to object recognition. Combining MRI-based diffusion-weighted imaging tractography with cortico-cortical paired associative stimulation protocol (ccPAS), we causally manipulated V1-to-IPS dorsal stream connectivity by strengthening synaptic efficacy between targeted regions through Hebbian-like plasticity mechanisms. Thirty-two healthy participants underwent three tractography-guided ccPAS sessions targeting right V1-to-IPS connectivity with different interstimulus intervals (9 ms, 0 ms, 30 ms). Before and after each session, participants performed a two-alternative forced-choice task requiring discrimination of either global shape or local features. Results showed that the critical 9 ms V1-to-IPS ccPAS protocol improved global shape discrimination, as indexed by accuracy and d′, relative to the 0 ms control condition, with no effect on reaction times or response bias. Although task specificity was not fully supported, exploratory analyses revealed no changes in local feature discrimination. These findings provide causal evidence that strengthening dorsal V1-to-IPS connectivity enhances global shape processing, supporting a direct contribution of the dorsal stream to object recognition and potentially guiding the development of targeted rehabilitative interventions after visual system damage.

## 1 Introduction

Human understanding of the physical world is deeply grounded in conscious perceptual experience, which is primarily visual. The brain’s ability to quickly and accurately identify objects by sight is crucial for survival and allows us to interact effectively with our environment. Nonetheless, disentangling the complex neural dynamics underlying object recognition, a crucial aspect of conscious visual processing, poses a significant challenge in contemporary cognitive neuroscience. Both animal and human models of visual information processing have thus far associated the successful recognition of objects with the ventral visual pathway (Goodale & Milner, 1992; Milner & Goodale, 2008; Mishkin & Ungerleider, 1982). However, novel evidence highlights a possible involvement of the dorsal stream in computing some key attributes crucial for visual object recognition (Ayzenberg et al., 2023).

According to the influential model proposed by Milner and Goodale, the visual system is segregated into two anatomically independent streams originating in the early visual cortex (Goodale & Milner, 1992). The ventral stream (also called vision-for-perception) projects to the inferotemporal cortex and is thought to support the perceptual identification of objects. The dorsal stream (also called vision-for-action) projects to the posterior parietal cortex and is thought to be involved in sensorimotor transformations for visually guided actions. Moreover, the dorsal stream receives direct magnocellular projections from the lateral geniculate nucleus (LGN) that bypass V1 (Sincich et al., 2004). Despite being grounded in a long history of neuropsychological, single-unit, behavioral, and functional imaging findings, this clear-cut distinction between the two pathways is currently under discussion. Visual processing, indeed, has turned out to be more tangled than initially postulated by this model. Consistently with this scenario, the hypothesis that the dorsal stream has a crucial role in object recognition by computing some identity-relevant object attributes like depth, orientation, structure-from-motion, and shape independently of goal-directed actions has been supported by recent compelling findings (Ayzenberg & Behrmann, 2022a, 2022b; Erlikhman et al., 2018; Freud et al., 2016, 2020; Xu, 2018). Importantly, according to this perspective, the dorsal stream is thought to be directly involved in object recognition, and its role is not simply redundant with respect to the information processed in the ventral stream. In keeping with this hypothesis, neuropsychological literature documented several cases of object recognition deficits (i.e. agnosia) in patients with parietal damage (Dalrymple et al., 2007; Karnath et al., 2000; Riddoch et al., 2008; Riddoch & Humphreys, 1987; Thomas et al., 2012; Warrington & Taylor, 1973) and accumulating evidence of different nature, such as lesion studies in non-human primate (Tsutsui et al., 2001; Van Dromme et al., 2016), human neuroimaging results (Bracci & Op de Beeck, 2016; Freud et al., 2017; Georgieva et al., 2008; Konen & Kastner, 2008; Vaziri-Pashkam & Xu, 2019), neural network models (Ayzenberg & Behrmann, 2022a), and causal studies with transcranial magnetic stimulation (Bagattini et al., 2015; Mazzi et al., 2014; Romei et al., 2011; Zachariou et al., 2017; Zaretskaya et al., 2013), supports the effector-independent perceptual representations in the dorsal stream.

In this scenario, the specific functional contribution of the dorsal stream to object recognition is controversial. Among the distinct operations involved in the complex process of visual object recognition, the ability to concurrently code the global shape with the local features of an object represents a necessary stage to appreciate its identity (Humphreys & Riddoch, 2006). Currently, a lively debate on object shape processing is unfolding between two opposite standpoints. On the one hand, the original proposal postulates that shape representation is uniquely supported by the ventral pathway without requiring any dorsal stream involvement (Goodale & Milner, 2023a, 2023b). On the other hand, this main stream conceptualization is challenged by the idea that global shape processing is computed in the dorsal stream and just later conveyed to the ventral stream (Ayzenberg et al., 2023; Ayzenberg & Behrmann, 2022a, 2022b, 2023; Bracci & Op de Beeck, 2016; Freud, Plaut, & Behrmann, 2016).

A direct approach to causally probe the contribution of the dorsal stream in object shape recognition is to manipulate its synaptic strength by means of cortico-cortical paired associative stimulation (ccPAS). ccPAS is a dual-site stimulation protocol in which pairs of transcranial magnetic stimulation (TMS) pulses are repeatedly delivered to two interconnected cortical regions at a specific time interval (i.e., inter-stimulus interval; ISI). ccPAS relies on Hebbian plasticity principles (Hebb, 1949) that postulate that the repeated activation of a presynaptic cell A immediately before activation of postsynaptic cell B induces synaptic strengthening (i.e., long-term potentiation; LTP). ccPAS protocols are thought to promote spike-timing-dependent plasticity (STDP) that is associated with changes in the strength of effective cortico-cortical connectivity between targeted regions that can be measured through modulations in behavioral performance on tasks supported by the stimulated connections. Previous studies testified these dynamics in motor areas (Buch et al., 2011; Casarotto et al., 2023; Chao et al., 2015; Chiappini et al., 2020; Fiori et al., 2018; Rizzo et al., 2011; Turrini et al., 2022; Veniero et al., 2013). Crucially, recent studies provided evidence of the possibility of inducing plasticity in the visual system and boosting visual perception according to STDP mechanisms (for a recent review see Tarasi et al., 2024). Pioneered by the first application of ccPAS to investigate plasticity within the visual system (Romei et al., 2016), several studies targeted the V1–V5 network, critical for motion awareness, consistently demonstrating that ccPAS aimed at strengthening reentrant V5-to-V1 pathway significantly improved visual motion perception (Bevilacqua et al., 2023; Chiappini et al., 2018; Di Luzio et al., 2022; Romei et al., 2016). By targeting the ventral visual pathway, Borgomaneri et al. (2023) investigated the role of this occipito-temporal network in face perception and showed that the strengthening of the superior temporal sulcus (STS)-to-V1 connections led to enhanced visual sensitivity to facial expressions. In the same vein, the protocol we propose here targets the right primary visual cortex (V1), that is, the starting point of the cortical visual pathways (Goodale & Milner, 1992), and the right intraparietal sulcus (IPS), a critical high-order key node of the dorsal visual stream that directly receives input from V1 (Greenberg et al., 2012). Notably, this parietal region has been demonstrated to show a selective activation, especially in the right hemisphere, for object–center part relations, which is an essential property for object recognition processes (e.g., Ayzenberg & Behrmann, 2022b; Goldstein-Marcusohn et al., 2024; Jeong & Xu, 2016; Zachariou et al., 2014). Recent findings (Wang et al., 2022) highlighted that the analysis of certain object properties computed in parietal regions might not require V1 when magnocellular input is available. Nevertheless, while subcortical projections may contribute to some aspects of visual object processing, this does not preclude the parallel involvement of the cortical pathway. This ccPAS protocol allows us to determine whether the dorsal stream’s role in global shape perception operates independently of other pathways, such as the ventral stream or subcortical magnocellular projections.

In this field, with high inter-individual variability in terms of observed effects being a critical issue, the selection of the cortical site to be targeted by TMS is of extreme importance. The vast majority of previously available studies adopted a “one-site-fits-all” targeting approach (Cash & Zalesky, 2023), which does not account for the substantial anatomical and functional brain variability that most likely affects the TMS efficacy (Cash et al., 2021; Menardi et al., 2022b; Opitz et al., 2016). Critically, the implementation of personalized spatial targeting highlighted the superiority of individualized TMS approaches by maximizing network engagement (Lynch et al., 2022; Menardi et al., 2022a, 2022b). This evidence is further supported by additional studies on the therapeutic application of TMS (Bagattini et al., 2021; Cash & Zalesky, 2023; Lynch et al., 2022).

In this perspective, advanced neuroimaging techniques, such as diffusion-weighted imaging (DWI) using MRI, allow for in vivo human brain fiber tracking. Currently, DTI (diffusion tensor imaging), originally formulated by Basser et al. (1994), represents the most commonly used model for investigating white-matter (WM) brain structure. It assesses the preferred direction of water diffusion along WM fiber tracts, known as anisotropy. Moreover, DTI allows the reconstruction of cerebral connectivity in vivo with the help of fiber tractography algorithms (Corbo et al., 2014). Crucially, TMS-induced signal propagation has been proven to engage structurally rather than functionally connected regions preferentially (Bortoletto et al., 2015; Momi et al., 2021).

Building on this, the approach we propose here capitalizes on the joint use of a ccPAS protocol (targeting the visual domain) and tractography measures characterizing the cortical and subcortical architecture at the individual level. For the first time, indeed, a personalized targeting approach driving the ccPAS protocol is introduced. Specifically, TMS targeting was individually guided by the identification of specific WM tracts with the electric field focalized on their cortical projections. This approach not only accounts for interindividual differences (Reisch et al., 2022) but also improves TMS spatial resolution by effectively and accurately recruiting specific subcortical structures (Nummenmaa et al., 2014) with a potential key impact on ccPAS plasticity effects.

**Fig. 1.**
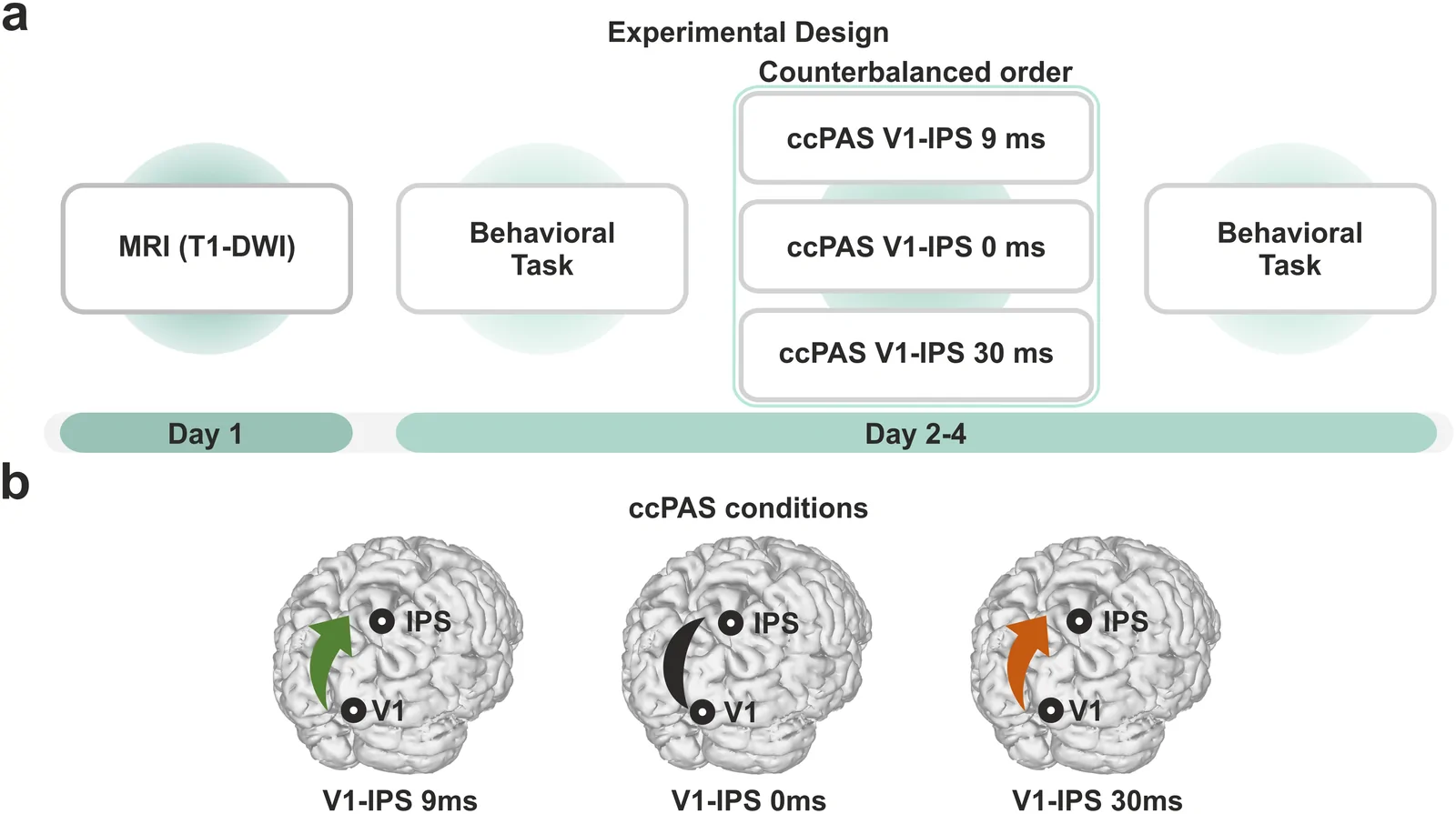
Experimental design. (a) Schematic overview of the experimental design procedure. It consisted of four experimental sessions divided into as many days. Firstly, an MRI acquisition was acquired (T1 and DWI sequence). In the last three sessions, participants underwent three ccPAS protocols delivered in a counterbalanced order across participants. Before and immediately after the ccPAS protocol, a two-alternative forced-choice match-to-sample task was delivered. (b) The three different ccPAS conditions. Each participant took part in all the conditions on three different days. Each session consisted of 90 paired pulses delivered at a rate of 0.1 Hz for 15 minutes, at 120% of the individual resting motor threshold (rMT). On the left, the V1–IPS 9 ms condition, which is expected to modulate the dorsal stream by stimulating right V1 (i.e., first TMS pulse) and right IPS (i.e., second TMS pulse) with an inter-stimulus interval (ISI) of 9 ms, is represented. In the middle, the V1–IPS 0 ms condition (first control condition), planned to account for possible nonspecific effects induced by the TMS. The TMS pulses were delivered simultaneously over the right V1 and right IPS (ISI 0 ms); according to the Hebbian principle, no modulation is expected here. On the right, the V1–IPS 30 ms condition (second control condition), designed to prove the time specificity of the effect, is represented. The right V1 and right IPS were targeted with an ISI of 30 ms. Arrows (when present) indicate the direction of the ccPAS-induced plasticity for the experimental (green) and control (orange) conditions.

The main aim of the present work is to investigate the contribution of the dorsal visual stream in object recognition by strengthening V1-to-IPS dorsal stream connectivity through an individualized tractography-based ccPAS protocol. Specifically, we employed a multimodal integrative approach (see Fig. 1) that takes advantage of the integration of a causal manipulative approach (i.e., ccPAS) with anatomo-structural (i.e., DTI) measures to delve into the role of the dorsal visual pathway in object global shape processing. According to our main hypothesis, if the dorsal stream is directly contributing to global shape processing, we expect to find an increase in the discrimination of global shape (H 1.1 – see Table 1) as a consequence of the ccPAS-induced strengthening of V1–IPS connectivity. The behavioral effects observed in the experimental V1–IPS ccPAS condition were compared with that of two control ccPAS conditions in order to assess the temporal specificity of the effect and to control for potential confounding factors. Additional control conditions were conducted in order to test the functional specificity of the ccPAS behavioral effect (i.e., task specificity and attention-related processing).

**Table 1.** Design table.

Question	Hypothesis	Sampling plan (N, power analysis)	Analysis plan	Rationale for deciding the sensitivity of the test for confirming or disconfirming the hp	Interpretation given to different outcomes	Theory that could be shown wrong by the outcomes	Observed outcome
**Q1)** Does the strengthening of dorsal stream connectivity modulate object shape recognition?	**H1.1)** If the dorsal pathway is directly involved in object shape recognition, we expect to find a greater effect on behavioral performance in the global shape task after the experimental V1–IPS 9 ms ccPAS condition (as compared with the ccPAS control conditions) as a consequence of the strengthening of the dorsal stream connectivity. *(Outcome neutral test/positive control: nonspecific TMS effects and temporal specificity)*	Sample size calculation was performed with G*Power software (v. 3.1.9.7), with a power of 95% and a significance level of 5%. To estimate the power needed to detect the main effect of “ccPAS condition” (V1–IPS 9 ms; V1–IPS 0 ms; V1–IPS 30 ms), we considered the significant main effect of ccPAS condition on the difference in motion sensitivity threshold reported in the study by Di Luzio et al. (2022) (post ccPAS-baseline; F2,48 = 6.51, p = 0.003; partial eta square = 0.21) and tested for a within-factors repeated measures ANOVA. The estimated sample size resulted in 32 participants (critical F = 3.145; actual power = 0.953). The resulting sample size estimation is consistent with a recent meta-analysis on ccPAS studies targeting the visual system (Di Luzio et al., 2024).	Repeated measures ANOVA on baseline-corrected (post-ccPAS–pre-ccPAS) accuracy, RTs, d’ values of “Global shape” trials with “ccPAS condition” (3 levels: V1–IPS 9 ms; V1–IPS 0 ms; V1–IPS 30 ms), and “Incongruency” (2 levels: “Totally incongruent” trials; “Partially incongruent” trials) as within-subjects factors.	Since no studies tested the efficacy of V1–IPS ccPAS on shape recognition, the effect size was determined based on a recent study using ccPAS to induce plastic changes in the visual pathways in order to investigate the effects on perceptual sensitivity (Di Luzio et al., 2022). In that study, the main effect of the ccPAS condition and statistical analysis are similar to our H1.1.	A significant “ccPAS condition” main effect showing a greater effect on discriminating global shape after V1–IPS 9 ms ccPAS compared with the control ccPAS protocols will support the involvement of the dorsal stream in global shape representation. V1–IPS 0 ms ccPAS protocol will represent a control condition accounting for possible *nonspecific TMS effects*. Indeed, 0 ms ccPAS condition is largely adopted as a control condition known to have no effects on behavioral performance. Furthermore, *temporal specificity* will be controlled by stimulating the same targets using a 30 ms ISI. The absence of a significant “ccPAS condition” main effect will not confirm H1.1 and H1.2 and H1.3 will not be tested. The absence of a significant “Incongruency” main effect will support the appropriateness of our task.	A significant “ccPAS condition” main effect showing a greater effect on discriminating global shape after V1–IPS 9 ms ccPAS compared with the control ccPAS protocols would pose evidence in contrast with the view that object shape representation is uniquely supported by the ventral pathway without requiring any dorsal stream involvement (Goodale & Milner, 2023a, 2023b), and would support the direct contribution of the dorsal stream in object shape recognition (Ayzenberg & Behrmann, 2022a, 2022b; Ayzenberg et al., 2023; Ayzenberg & Behrmann, 2023; Bracci & Op de Beeck, 2016; Freud et al., 2016).	H1.1 was supported by a significant “ccPAS condition” main effect on baseline-corrected accuracy and d’ (not on RTs) with post hoc comparisons indicating that V1–IPS 9 ms led to a greater improvement with respect to the V1–IPS 0 ms (control condition).
	**H 1.2)** The effect on behavioral performance for the discrimination of global shape after the experimental V1–IPS 9 ms ccPAS protocol is not due to a change in participants’ response bias. *(Outcome neutral test/positive control: effect on response bias)*	Sample size calculation has been performed considering H1.1.	Repeated measures ANOVA on baseline-corrected (post-ccPAS–pre-ccPAS) response criterion c values of “Global shape” trials with “ccPAS condition” (3 levels: V1–IPS 9 ms; V1–IPS 0 ms; V1–IPS 30 ms) as within-subjects factor.	The effect size for power analysis was determined considering H1.1.	A significant “ccPAS condition” main effect showing a greater shift in response criterion when discriminating global shape after V1–IPS 9 ms ccPAS compared with the control ccPAS protocols will NOT sustain H1.2. In this case, the effect of response criterion’s shift on the primary outcome measures (accuracy, RTs, and d’) and how this shift may impact the interpretation of H1.1 will be discussed.	-	H1.2 was confirmed by the lack of a significant “ccPAS condition” main effect on baseline-corrected response criterion.
	**H1.3)** If the dorsal pathway is directly involved in object shape recognition, after the experimental V1–IPS 9 ms ccPAS protocol, we expect to find a greater effect on behavioral performance for the discrimination of global shape as compared with the discrimination of local features. *(Outcome neutral test/positive control: Task specificity and attention-related processing)*	Sample size calculation has been performed considering H1.1.	Repeated measures ANOVA on baseline-corrected (post-ccPAS–pre-ccPAS) accuracy, RTs, and d’ values with ccPAS condition” (3 levels: V1–IPS 9 ms; V1–IPS 0 ms; V1–IPS 30 ms), “Task” (2 levels: “Global shape task” and “Local features task”), and “Incongruency” (2 levels: “Totally incongruent” trials; “Partially incongruent” trials) as within-subjects factor.	The effect size for power analysis was determined considering H1.1.	A statistically significant difference showing a greater improvement in global shape discrimination as compared with local feature discrimination will support the *task specificity* of the V1–IPS 9 ms ccPAS protocol. As the dorsal stream is also known to play an important role in visual attention (Corbetta & Shulman, 2002), task specificity will allow to rule out the possibility that the improvement in global shape discrimination is due to attention-related processing modulation. The absence of a statistically significant difference could be interpreted and will be discussed as possible: involvement of the dorsal stream in both global shape and local features representation; -evidence of the fact that local features have been computed as global shape; -evidence of an attentional/arousal effect due to IPS potentiation. The absence of a significant “Incongruency” main effect will support the appropriateness of our task.	A statistically significant difference showing a greater improvement in global shape discrimination as compared with local feature discrimination in our critical condition would pose evidence in contrast with the view that object shape representation is uniquely supported by the ventral pathway without requiring any dorsal stream involvement (Goodale & Milner, 2023a, 2023b), and would support the direct contribution of the dorsal stream in object shape recognition (Ayzenberg & Behrmann, 2022a, 2022b; Ayzenberg et al., 2023; Ayzenberg & Behrmann, 2023; Bracci & Op de Beeck, 2016; Freud et al., 2016).	H1.3 was unsupported by pre-registered analyses, since no significant “ccPAS condition x Task” interaction was found. However, exploratory analyses showed no significant main effect of “ccPAS condition” on local features task for both accuracy and d’.

## 2 Methods

This manuscript corresponds to Stage 2 of a Registered Report. The Stage 1 protocol has been peer reviewed and is available at https://osf.io/2naqs.

### 2.1 Ethics information

Ethical approval for this study was obtained from the local ethical committee (Comitato Etico Territoriale Area Sud-Ovest Veneto - CET-ASOV; approval number 371CET). Written informed consent was obtained from all participants before data collection. They were reimbursed for their participation on an hourly basis. The study was performed in accordance with the ethical standard of the 2013 Declaration of Helsinki. Participants were recruited through word of mouth, as well as through a combination of printed and electronic advertisements displayed on notice boards at the University of Verona, where the study was conducted (Perception and Awareness (PandA) laboratory).

### 2.2 Sample size estimation

Sample size calculation was performed with G*Power software (v. 3.1.9.7), with a power of 95% and a level of significance of 5%. The sample size estimation was performed for the primary research question (Q1: Does the strengthening of dorsal stream connectivity modulate global shape representation?) and specifically on H1.1, which aims at investigating the effect of V1–IPS 9 ms ccPAS protocol on global shape discrimination as compared with control ccPAS protocols (i.e., V1–IPS 0 ms and V1–IPS 30 ms). Because in the literature no studies tested the efficacy of V1–IPS ccPAS on shape discrimination, the sample size was determined based on a recent study using ccPAS to induce plastic changes in the visual pathways in order to investigate the effects on perceptual sensitivity (Di Luzio et al., 2022). To estimate the power needed to detect the main effect of within-subjects “ccPAS condition” (V1–IPS 9 ms; V1–IPS 0 ms; V1–IPS 30 ms) on baseline-corrected (post-ccPAS–pre-ccPAS) behavioral variables, we considered the significant main effect of ccPAS condition on the difference in motion sensitivity threshold in Di Luzio and colleagues paper (post ccPAS-baseline; F(2,48) = 6.51, p = 0.003; partial eta square = 0.21) and tested power for a within-factors repeated measures ANOVA. The estimated sample size resulted in 32 participants (critical F = 3.145; actual power = 0.953). The resulting sample size estimation is consistent with a recent meta-analysis on ccPAS studies targeting the visual system (Di Luzio et al., 2024).

### 2.3 Exclusion criteria

To take part in the study, participants had to be above 18 and below 35 years of age and show normal or corrected-to-normal visual acuity in both eyes. Participants were excluded if left handed according to a standard handedness inventory (Edinburgh Handedness Questionnaire; Oldfield, 1971), if they had any history of neurological or psychiatric diseases, or if they reported any contraindications to MRI and TMS (Rossi et al., 2021). Moreover, before being enrolled in the study, participants underwent a preliminary session of the behavioral task. To be considered eligible to participate in the study, participants had to show an accuracy above chance according to the binomial test (58.33% accuracy, n = 120, p = 0.041). After the recruitment, participants could be excluded from the analyses in the following cases: (1) they did not complete experimental sessions due to personal or technical issues (e.g., discomfort during TMS, hardware/software failures); (2) they were identified as outlier participants when showing mean reaction times (RTs) above the third quartile (Q3) plus 1.5*interquartile range (IQR) or below Q1 minus 1.5* IQR of the global RT distribution. Should an enrolled participant meet any exclusion criteria, another participant was recruited in order to reach a total sample size of 32 as outlined in the sampling plan (see Section 2.2).

### 2.4 Experimental procedure

A schematic overview of the study design is illustrated in Figure 1a. Eligible participants underwent an MRI acquisition (see Section 2.6) before the ccPAS administration. ccPAS protocols were delivered in three experimental sessions conducted on separate days, whose order was counterbalanced across participants. Sessions were performed with at least 7 days between them to prevent possible addictive effects of ccPAS (Koch et al., 2013; Sale et al., 2007; Veniero et al., 2013). At the beginning of each ccPAS session, neuronavigation procedures were carried out. Before and immediately after the ccPAS protocol, participants underwent a two-alternative forced-choice match-to-sample task (see Section 2.5). The experimental sessions differed only in the ccPAS protocol delivered.

### 2.5 Behavioral task

Participants were tested in a dimly illuminated test room before and after each ccPAS protocol with the same behavioral procedure. They sat in front of a 24 in. ASUS monitor (resolution 1920 x 1080, refresh rate 144 Hz) placed at a viewing distance of 57 cm, with the head laying on an adaptable chin rest to align the participant’s eyes to the center of the screen.

**Fig. 2.**
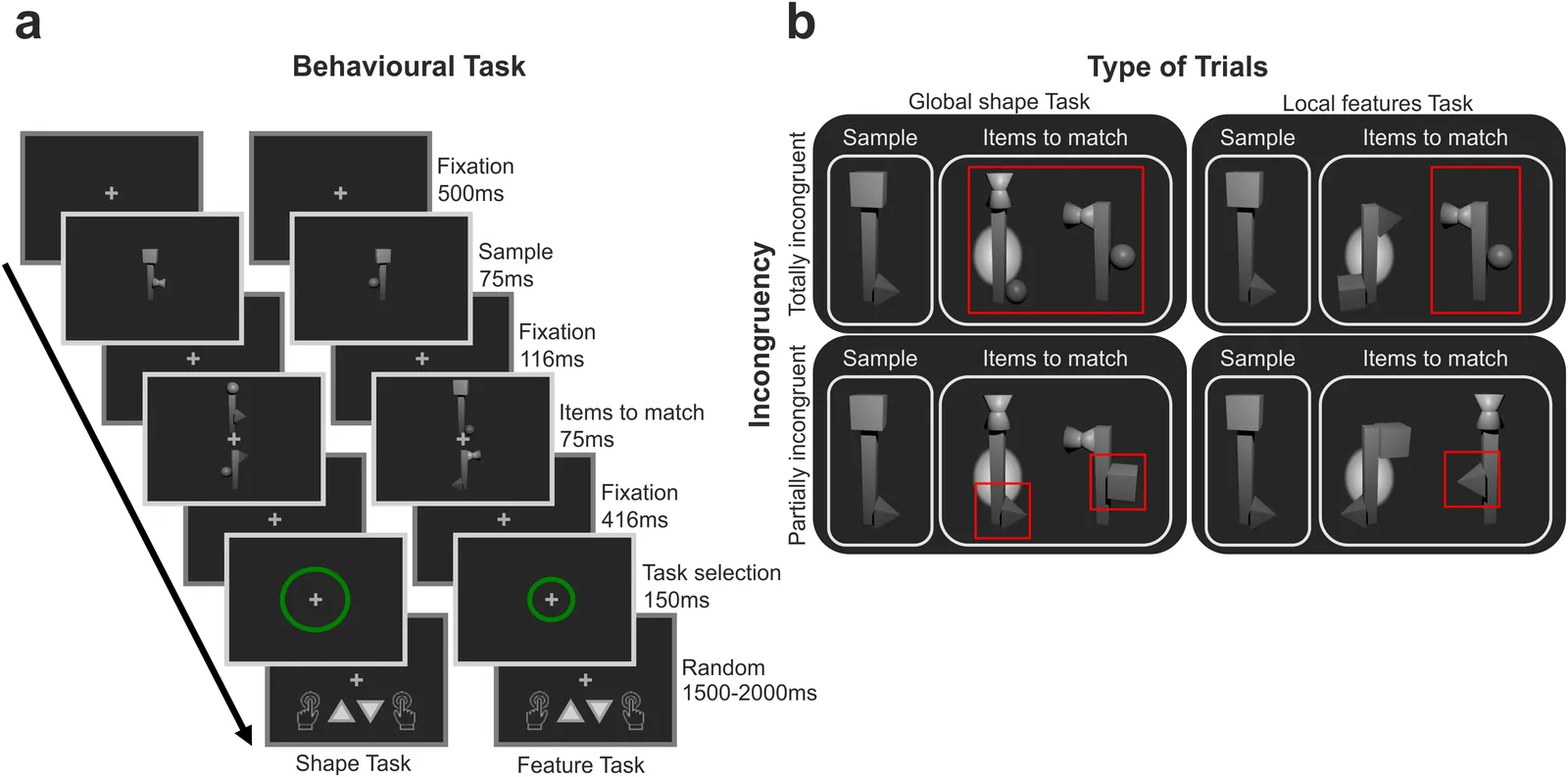
Behavioral task. (a) The trial structure of the modified two-alternative forced choice (2AFC) match-to-sample discrimination task. Participants were asked to report which of the two items displayed matched the sample previously seen by discriminating the global shape or the features of the objects. The task started by asking the participant to maintain the gaze on the fixation cross. After 500 ms, the sample appeared in the center of the screen for 75 ms. After 116 ms, two items to match appeared above and under the fixation cross for 75 ms. After an interval of 416 ms, around the fixation cross, a circle appeared for 150 ms revealing the task to be computed: a big green circle for the “Global shape task”, and a small green circle for the “Local features task.” For the response, a random interval lasting between 1500 and 2000 ms was left. (b) The properties of the different types of trials. For each task, trials could be divided into “Totally incongruent” trials and “Partially incongruent trials.” The incongruency was based on the presence of the features of the sample in the items to match. In totally incongruent trials of the global shape task, the items to match had all the features different from the sample, while in the local features task, only the incorrect item had all the features different from the sample. In partially incongruent trials of the global shape task, one feature in each item or in only one item could be shared with the sample, while in the local features task, only the incorrect item could share one feature with the sample. The correct response is highlighted in white. The red square highlights the critical properties that characterize the incongruency of the items-to-match with the sample (“Totally incongruent trials” vs “Partially incongruent trials”).

A modified two-alternative forced choice (2AFC) match-to-sample discrimination task was employed (see Fig. 2a) (adapted from Chouinard et al., 2017). Visual stimuli consisted of 3D elongated geons composed of parts with different local features (see Fig. 2b) that can be arranged in several spatial configurations and shown from several observation points (modified from a previously used stimuli dataset, Ayzenberg & Behrmann, 2022b). Those stimuli are chosen to facilitate a response along the dorsal stream, due to their properties in evoking a visuo-motor response (Freud et al., 2020; Marini et al., 2019) and due to the sensibility of the dorsal stream to changes in visual stimuli (James et al., 2002; Valyear et al., 2006) independently from task demands. This is essential for our protocol since the effectiveness of the ccPAS procedure also depends on the engagement of the network of interest. Participants were asked to specify which of two stimuli matched the sample stimulus in terms of spatial arrangement (hereafter called “global shape task”) or in terms of local features (hereafter called “local features task”). Pilot data (see Supplementary Information—Pilot data and Supplementary Fig. S1 ) demonstrated that the two tasks were comparable in terms of difficulty since no differences between the global shape task and the local features task were found in accuracy, RTs, and d’.

Each behavioral session comprised 1 training block of 36 trials and 8 experimental blocks of 36 trials each for a total of 288 trials (120 “Global shape task” trials, 120 “Local features task” trials, 24 “Global shape task” catch trials, and 24 “Local features task” catch trials). The entire behavioral session lasted ~21 minutes.

In each trial, one sample and two items-to-match were presented. In the “Global shape task” trials, items-to-match could be either totally incongruent with the local features of the sample (i.e., all items’ local features were different from the sample’s local features), or partially incongruent (i.e., at least one item shared a feature with the sample). In the “Local features task” trials, both the items-to-match had a different global shape than the sample, and the incorrect item could be either totally incongruent with the features of the sample or partially incongruent, sharing only one feature with it.

Each trial began with the presentation of a central fixation cross on a dark gray screen for 500 ms (1.62 cm/m²), then a central stimulus (sample image) was presented for 75 ms (2.3° x 4.3°; 8.03 cm/m²) on the fixation cross. After an interval of 116 ms (in order to avoid the fusion of the stimuli), two central items-to-match were presented for 75 ms above and under the fixation cross at 4.5° eccentricity from central fixation. After 416 ms, a central green circle was displayed for 150 ms to inform the participants about the task to be performed: a large green circle (8.4° in diameter, matching the size of the global target) indicated the “Global shape task,” while a small green circle (2.8° in diameter, corresponding to the dimension of the local features) indicated the “Local features task”. After the circle onset, participants were asked to report as fast and accurately as possible which stimulus was the one that shared with the sample image the characteristic required in the task (i.e., global shape or local features) by pressing two vertical keys on a response box, standing for the upper or above stimulus. A variable randomized interval, lasting between 1500 and 2000 ms, was left for the response before the start of the next trial. The position of the correct item-to-match was randomized across trials. The order of trials (i.e., “Global shape” task and “Local features” task) was randomized within each block. Different local features and different spatial configurations of objects were balanced across trials considering both sample images and items-to-match. Participants rested for 30 s during inter-block intervals until a 3-s countdown highlighted the beginning of the following block. The response was delivered by using the left and the right index finger, respectively, on the two vertical keys. The position of the fingers was reversed halfway through the task and the starting order of fingers was balanced across participants. Both RTs and accuracy (percentage of correct responses) were recorded. The stimuli duration and the intervals between them were tested by means of a photodiode and an oscilloscope.

Before starting, participants were instructed about the type of stimuli used (i.e., elongated geons) and the structure of the trial sequence. Some examples of the two types of tasks were presented to elucidate the definition of global shape and local features and to describe the task selection by means of the two different circles (large green for global shape and small green for local features, respectively).

### 2.6 MRI acquisition

All participants underwent an MRI session before the ccPAS sessions. Anatomical images were acquired with a 3T Philips Elition S with a 32-channel head RF receive coil. MR images included (i) 3D T1-weighted (T1w) Turbo-field echo image (1 mm-isotropic TE/TR = 3.8/8.4 ms, TI = 1050 ms), (ii) diffusion-weighted (DWI) data (2 mm-isotropic, TE/TR = 126/6000 ms, shells: b = {0,1000,2000} s/mm, 12/32/64 directions).

### 2.7 MRI pre-processing

All participant T1w images went through standard preprocessing steps: (I) raw DICOM T1-w images were converted to NifTI format using dcm2niix software (Li et al., 2016); (II) AC-PC (Anterior-Posterior Commissures) alignment was performed by means of rigid registration to the MNI152 T1-w template (Avants et al., 2008); (III) brain mask was estimated (Avants et al., 2014) and used to perform the Bias-Field Correction restricted on brain voxels only (N4-Bias Field Correction tool; Tustison et al., 2010); (IV) segmentation into six brain tissues was performed with several algorithms (Amorosino et al., 2020; Çiçek et al., 2016; Ronneberger et al., 2015; Smith et al., 2012) to then establish the best performing one for accurate estimation of the tractogram given peculiarities of dorsal/occipital regions); (V) finally, a synthetic T2-w image was created using AFNI toolkit (Cox, 1996).

DWI data were preprocessed using combinations of MRtrix3 commands (Tournier et al., 2012) in conjunction with FSL (Jenkinson et al., 2012). Images were corrected for thermal noise, Eddy currents, and Gibbs ringing. By applying diffeomorphic registration to the susceptibility-induced echo-planar imaging (EPI) of the DWIs volumes, and by using a combination of data from an undistorted structural MRI and DWI reversed-phase encoding directions (i.e., AP and PA acquisitions), distortion correction was introduced. Further corrections were applied to control for bias field inhomogeneities on DWI data.

Reconstructions of streamlines were carried out using the MRtrix3 program (Tournier et al., 2012). The WM fiber Orientation Distribution Function (fODF) was obtained from the estimation of the response function using the Dhollander method (Dhollander et al., 2016) by applying the multi-shell, multi-tissue Constrained Spherical Deconvolution (CSD, Jeurissen et al., 2014) (lmax = 6; threshold = 0.5). In order to confine the streamlines’ length between 20 and 250 millimeters and to halt the tracking process after choosing at least 10 million streamlines, probabilistic anatomically constrained tractography, or ACT, was calculated using CSD (cutoff 0.06). The SIFT2 technique was then used to guarantee that the tractogram was correctly filtered and to counteract overfitting (Smith et al., 2015).

### 2.8 Tract dissection and neuronavigation

**Fig. 3.**
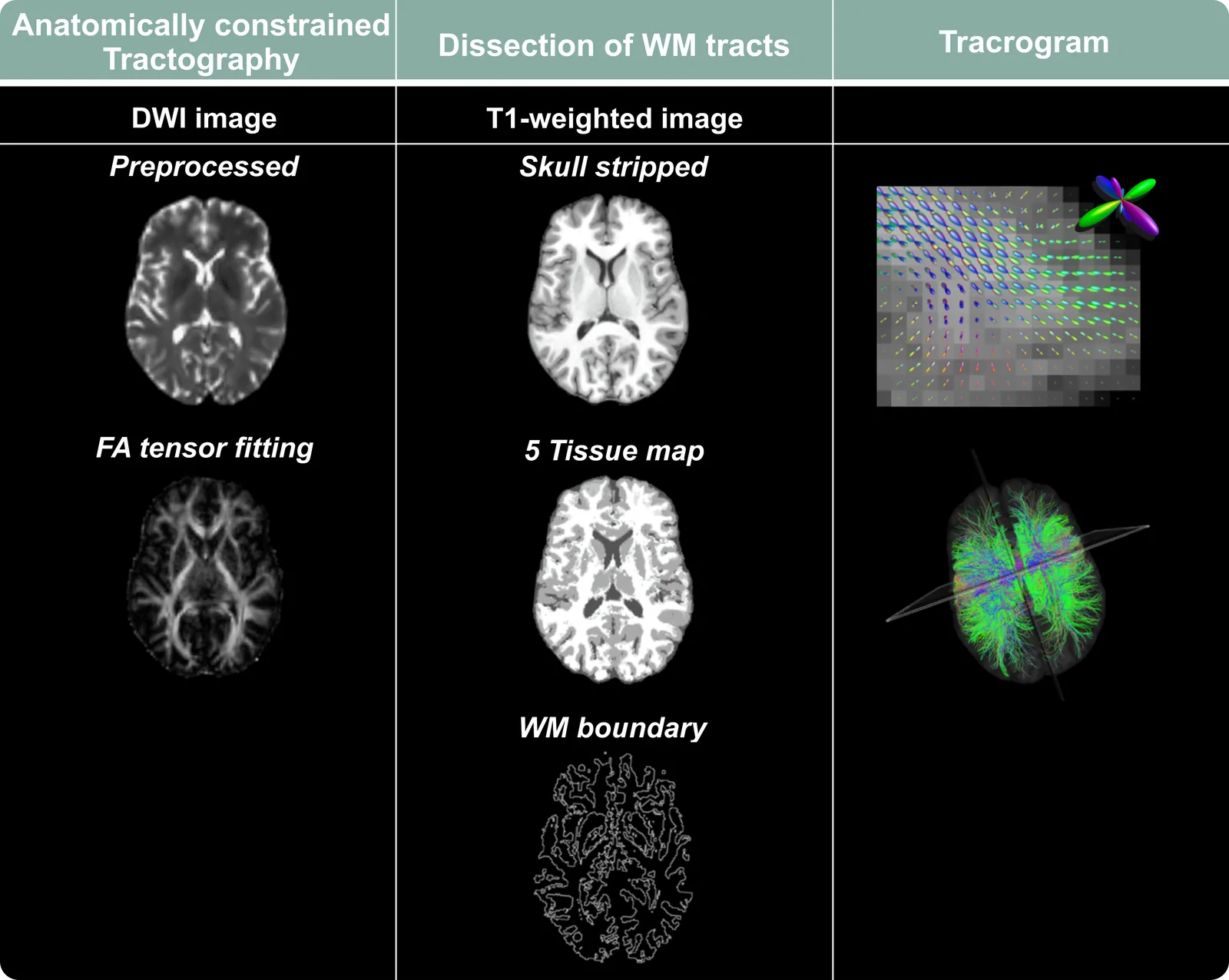
DWI Pipeline. Description of relevant pre-processing and fitting steps of MRI processing are reported. Data from a single subject are presented: (i) In the first column, DWI preprocessed images b0-images and tensor DTI (diffusion tensor imaging) fitting FA (fractional anisotropy) maps are displayed; (ii) in the second column, T1-weighted skull-stripped pre-processed image and segmentation results are displayed together with the representation of the white-matter (WM) boundary; (iii) visual representation of FOD (fiber orientation distribution) is reported (Tournier et al., 2019) together with the tractogram at the individual level derived from the anatomically constrained tractography (ACT).

Once the fully masked tractogram was obtained, it was processed in TrackVis to dissect the V1–IPS tract of interest, representing the dorsal stream. Seed regions of interest (ROIs) were identified based on a combination of anatomical and atlas-based approaches to ensure precision and reproducibility. First, the IPS ROI was anatomically localized using individual T1-weighted images. To account for cortical variability across participants, we co-registered the Wang atlas regions (IPS1-IPS2-IPS3; Wang et al., 2015) to each subject’s native space. This atlas provides a reliable anatomical framework for identifying IPS regions. By overlaying the atlas on individual brains, we ensure that the identified ROIs are contained within the predefined IPS regions from the atlas. This step minimized user-related variability and provided consistency in anatomical placement. While anatomical localization was prioritized, we acknowledge the importance of functional definitions in cases of significant cortical variability, as highlighted by Ayzenberg & Behrmann (2022b). After atlas-based co-registration, manual inspection was performed to refine ROI placement based on individual anatomical variations visible in T1-weighted images. This ensured that seed regions are accurately positioned for tractography while adhering to anatomical landmarks. The identified cortical origins of the bundles (Tagliaferri et al., 2023) were then used as targets for ccPAS protocols by means of a neuronavigation system (SofTaxic software v.3.4, EMS, Italy). Individual T1w images were used for stereotaxic frameless neuronavigation, by means of an optical infrared camera and reflective markers placed on the participant’s head and TMS coil. To obtain population-level localization of the stimulation sites, the individual brains were then normalized in MNI space, and target coordinates were extracted (Fig. 3). A visual representation of the anatomical dissection of the WM bundle of interest for tractography-informed neuronavigation in a pilot subject is provided in Supplementary Figure S2.

### 2.9 TMS parameter definition

Stimulation intensity was defined according to the individual resting motor threshold (rMT), which was determined at the beginning of each session. Electromyographic activity was visualized online by means of a bipolar belly–tendon montage of the first dorsal interosseous (FDI) muscle of the left hand using Ag/AgCl pre-gelled surface electrodes. The active recording electrode was placed over the muscle belly, and the reference electrode on the proximal phalanx bone of the index finger. The ground electrode was placed over the forearm. The motor hotspot of the left FDI muscle was identified in the right hemisphere as the area eliciting the highest and most reliable motor-evoked potentials (MEPs) with the same TMS intensity by moving the coil in 0.5 cm steps around the motor hand area. Then, the individual rMT was defined as the minimum TMS intensity (expressed as the percentage of maximum stimulator output) able to elicit an MEP of at least 50 µV by employing the parameter estimation by sequential testing (PEST) procedure (Awiszus, 2003; Dissanayaka et al., 2018) (i.e., maximum-likelihood threshold-hunting approach). The coils were placed tangentially on the surface of the scalp. V1 stimulation on the right hemisphere was performed by placing the coil at an angle of 120 degrees (Chiappini et al., 2018; Di Luzio et al., 2022; Romei et al., 2016). For the right IPS stimulation, the coil was oriented with an angle of about 60 degrees counterclockwise, thus maintaining the same orientation between the two coils (Di Luzio et al., 2022). Neuronavigation software (SofTaxic, E.M.S., Bologna, Italy) combined with a 3D optical digitizer (Polaris Vicra, NDI, Waterloo, Canada) allowed us to save the coil positions for the ccPAS sessions.

### 2.10 ccPAS protocols

Participants underwent an experimental ccPAS protocol, targeting dorsal stream connectivity (V1–IPS 9 ms), and two control ccPAS protocols, to control for the nonspecific TMS effects (V1–IPS 0 ms) and the temporal specificity (V1–IPS 30 ms) of the protocol (see Fig. 1b). The order of the ccPAS protocols was counterbalanced across participants.

The ccPAS protocols were delivered using two 50 mm custom-made figure-of-eight coated coils connected to a Magstim BiStim² stimulator (Magstim Company, UK). In total, 90 paired pulses were delivered at a rate of 0.1 Hz for a total duration of about 15 minutes, with each pair consisting of 2 monophasic transcranial magnetic pulses^1^ (Bevilacqua et al., 2023; Borgomaneri et al., 2023; Chiappini et al., 2022; Fiori et al., 2018; Turrini et al., 2023). TMS intensity was set at 120% of the individual resting motor threshold (rMT) for both conditioning and test pulses as done in previous ccPAS studies targeting parietal areas (Casula et al., 2016; Guidali et al., 2023; Nord et al., 2019; Santarnecchi et al., 2018; Zibman et al., 2019). Neuronavigation software (SofTaxic, E.M.S., Bologna, Italy) combined with a 3D optical digitizer (Polaris Vicra, NDI, Waterloo, Canada) was used throughout the experiment to maintain the coils’ position over the participant’s head within a 2-mm accuracy threshold. Participants were asked to maintain their gaze on a central purple fixation cross and to mentally count the number of times it changed into yellow (i.e., 10 times during the protocol) to avoid sleepiness and keep their attention high during the ccPAS—a critical condition for protocols’ effectiveness (Stefan et al., 2000). TMS pulses and trial presentation were controlled with E-Prime 3.0 (Psychology Software Tools Inc., Pittsburgh, PA, USA).

#### 2.10.1 Experimental ccPAS protocol targeting dorsal stream connectivity from V1 to IPS (V1–IPS 9 ms)

TMS pulses were delivered over V1, unanimously considered as the origin of the dorsal stream, and IPS, a critical parietal cortical region along the dorsal stream. Both pulses were delivered over the right hemisphere. Indeed, right IPS has been demonstrated to be engaged in processing object properties relevant for recognition, and an increasing body of evidence shows a selective activation for object–center part relations (Ayzenberg & Behrmann, 2022b; Bracci & Op de Beeck, 2016; Freud et al., 2016; Goldstein-Marcusohn et al., 2024; Jeong & Xu, 2016; Zachariou et al., 2014). The stimulation sites (i.e., right V1 and right IPS) were identified using tractography-based individualized cortical sites (see Section 2.7).

Coils’ orientation is described above (see Section 2.9). The conditioning TMS pulse (i.e., first) was delivered over V1, while the test TMS pulse (i.e., second) was delivered over the IPS with an ISI of 9 ms. The critical ISI has been chosen based on a previous electrocorticographic (ECoG) recording study aimed at estimating the spatiotemporal dynamics of visual information processing along the human posterior cortex (Regev et al., 2018).

#### 2.10.2 Control ccPAS protocol targeting dorsal stream connectivity from V1 to IPS (V1–IPS 0 ms)

In order to test possible nonspecific effects induced by the TMS, a control ccPAS protocol with ISI 0 ms was employed. In this protocol, the target areas were the same as in V1–IPS 9 ms condition but the pulses were delivered simultaneously (ISI = 0 ms). According to the Hebbian principles (Hebb, 1949), synaptic efficacy increases if the presynaptic neuron persistently takes part in the firing of the postsynaptic target neuron. However, if two neurons fire at the same time, then one cannot have caused or taken part in the firing of the other (Caporale & Dan, 2008; Romei et al., 2016). Thus, no net spike timing-dependent plasticity is expected following the V1–IPS 0 ms ccPAS condition.

#### 2.10.3 Control ccPAS protocol dorsal stream connectivity from V1 to IPS (V1–IPS 30 ms)

In order to test the temporal specificity of the experimental V1–IPS 9 ms ccPAS condition, a control ccPAS protocol was employed. In this protocol, the target areas were the same as in the V1–IPS 9 ms condition, but the pulses were delivered with a 30 ms ISI. This ISI has been chosen on the basis of a previous study (Di Luzio et al., 2022) that tested the same connection in the opposite direction (IPS-V1), finding an improvement in metacognition.

### 2.11 Analysis plan

Statistical analyses were performed using Jamovi (Version 2.3.28; The Jamovi Project, 2024). Statistical significance was set at p<0.05.

For each participant, trials with RTs < 150 ms were considered unreliable answers, discarded, and not considered for further analysis.

Discrimination performance was indexed by the accuracy and RTs values. Both measures were calculated for each participant separately for “Global shape task” trials and “Local features task” trials, dividing “Totally incongruent” and “Partially incongruent” trials. The normality of accuracy and RTs data was assessed by means of the Shapiro–Wilk test. In case data would have not satisfied the properties of normal distribution, data would have been transformed according to the commonly used transformations of continuous data: square root, base-10 logarithmic, and inverse transformation. The transformation showing the best fit to a normal distribution would have been assessed through visual inspection of data distribution.

Additionally, in order to obtain a measure of accuracy not affected by the response bias, we calculated d’ sensitivity measure as assessed by signal detection theory (Macmillan & Creelman, 1991). d’ was calculated for each participant separately for “Global shape task” trials and “Local features task” trials, and for “Totally incongruent” and “Partially incongruent” trials, by subtracting the standardized false alarm rate of responses from the standardized hit rate as follows:

d’=z(Hitrate)–z(FalseAlarmrate).

These rates correspond to probabilities on the normal distribution, therefore, z(Hit rate) and z(False Alarm rate) are the z-scores that correspond to the normal distribution’s tail p-values represented by Hit and False Alarm. Task response accuracy in the modified 2AFC task was scored as in Borgomaneri et al. (2023). Correct responses were considered as follows: an “up” response when the correct item-to-match was presented at the top of the screen counted as a “hit,” whereas a “down” response when the correct item-to-match was presented at the bottom of the screen counted as a correct rejection. Incorrect responses were considered as follows: an “up” response when the correct item-to-match was presented at the bottom of the screen was counted as a false alarm, whereas a “down” response when the correct item was at the top of the screen was considered a miss. In order to further control for a possible response bias (outcome neutral test/positive control), the SDT criterion index c was calculated. The criterion index reflects the participants’ tendency to favor one response over another in a 2AFC discrimination task.

c was calculated for each participant for “Global shape task” trials, by averaging the standardized hit rate of responses and the standardized false alarm rate as follows:

$$c=-\frac{\left[z\left(Hit\;rate\right)+z\left(False\;Alarm\;rate\right)\right]}{2}.$$

Design Table 1 provides a detailed overview of the analyses for all the outlined research questions.

**Question 1. Does the strengthening of dorsal stream connectivity modulate global shape representation?**

**Hypothesis 1.1. If the dorsal pathway is directly involved in object shape recognition, we expect to find a greater effect on behavioral performance in the global shape task after the experimental V1**–**IPS 9 ms ccPAS condition as a consequence of the strengthening of the dorsal stream connectivity.**

In order to test whether strengthening dorsal pathway connectivity through V1–IPS 9 ms ccPAS protocol induces a greater effect in global shape discrimination performance as compared with control ccPAS conditions, separate repeated measures ANOVA on baseline-corrected (post-ccPAS–pre-ccPAS) accuracy, RTs, and d’ values of “Global shape task” trials with “ccPAS condition” (3 levels: V1–IPS 9 ms; V1–IPS 0 ms; V1–IPS 30 ms) and “Incongruency” (2 levels: “Totally incongruent” trials; “Partially incongruent” trials) as within-subjects factors was performed.

**Hypothesis 1.2. The effect on behavioral performance for the discrimination of global shape after the experimental V1**–**IPS 9 ms ccPAS protocol is not due to a change in participants’ response bias.**

In order to verify that the effect on discrimination performance after V1–IPS 9 ms ccPAS protocol is not due to a change in participants’ response criterion, a repeated measures ANOVA on baseline-corrected (post-ccPAS–pre-ccPAS) c values of “Global shape task” trials with “ccPAS condition” (3 levels: V1–IPS 9 ms; V1–IPS 0 ms; V1–IPS 30 ms) as within-subjects factor was performed.

**Hypothesis 1.3. If the dorsal pathway is directly involved in object shape recognition, after the experimental V1**–**IPS 9 ms ccPAS protocol, we expect to find a greater increase in behavioral performance for the discrimination of global shape as compared with the discrimination of local features.**

In order to verify the task specificity of the increase in discrimination performance after each ccPAS protocol (i.e., V1–IPS 9 ms; V1–IPS 0 ms; V1–IPS 30 ms), a repeated measures ANOVA was performed on both baseline-corrected (post-ccPAS–pre-ccPAS) accuracy, RTs, and d’ values of all trials (global shape task and local features task trials) with “Task” (2 levels: “Global shape task” and “Local features task”), “ccPAS condition” (3 levels: V1–IPS 9 ms; V1–IPS 0 ms; V1–IPS 30 ms), and “Incongruency” (2 levels: “Totally incongruent” trials; “Partially incongruent” trials) as within-subjects factors.

In all the statistical analyses so far reported (H1.1, H1.2, H1.3), the Greenhouse–Geisser correction was applied when appropriate. Partial eta squared was computed and reported as a measure of effect size for the main effects, whereas Cohen’s d was computed and reported for post hoc comparisons. To account for multiple comparisons, Bonferroni correction was applied when necessary.

## 3 Results

### 3.1 Sample description

Ninety-four healthy participants underwent the preliminary session of the behavioral task. Forty-seven participants performed below chance level in at least 1 of the 2 tasks: 16 participants were below chance level in both tasks (global shape: mean = 0.519, sd = 0.045; local features: mean = 0.526, sd = 0.039), 8 only in the global shape task (global shape: mean = 0.518, sd = 0.032; local features: mean = 0.626, sd = 0.039), and 23 only in the local features task (global shape: mean = 0.690, sd = 0.068; local features: mean = 0.523, sd = 0.042). According to pre-registered exclusion criteria, they were not further involved in the study. Two participants were excluded due to incompatibility with MRI; 2 were excluded because they met some TMS exclusion criteria; and 3 participants withdrew from the experiment before undergoing MRI acquisition. Forty participants performed above chance level and were, therefore, considered eligible for the experimental procedures. Eight participants were not included in the final sample for the following reasons: (i) 5 participants did not complete the experimental sessions due to personal issues; (ii) 2 participants were incidentally found to have a radiological abnormality on MRI; (iii) 1 participant did not adhere to the task instructions, performing only part of the behavioral task. Hence, the final sample included in the analyses comprised 32 participants (15 females; age 23.56 ± 3.60 years; education 16.22 ± 1.34; Edinburgh Handedness Questionnaire Laterality Quotient > 54.3). Detailed information on participants’ demographics, individual ccPAS target coordinates, and rMT values are provided in Supplementary Table S1.

### 3.2 Preregistered analyses

**Fig. 4.**
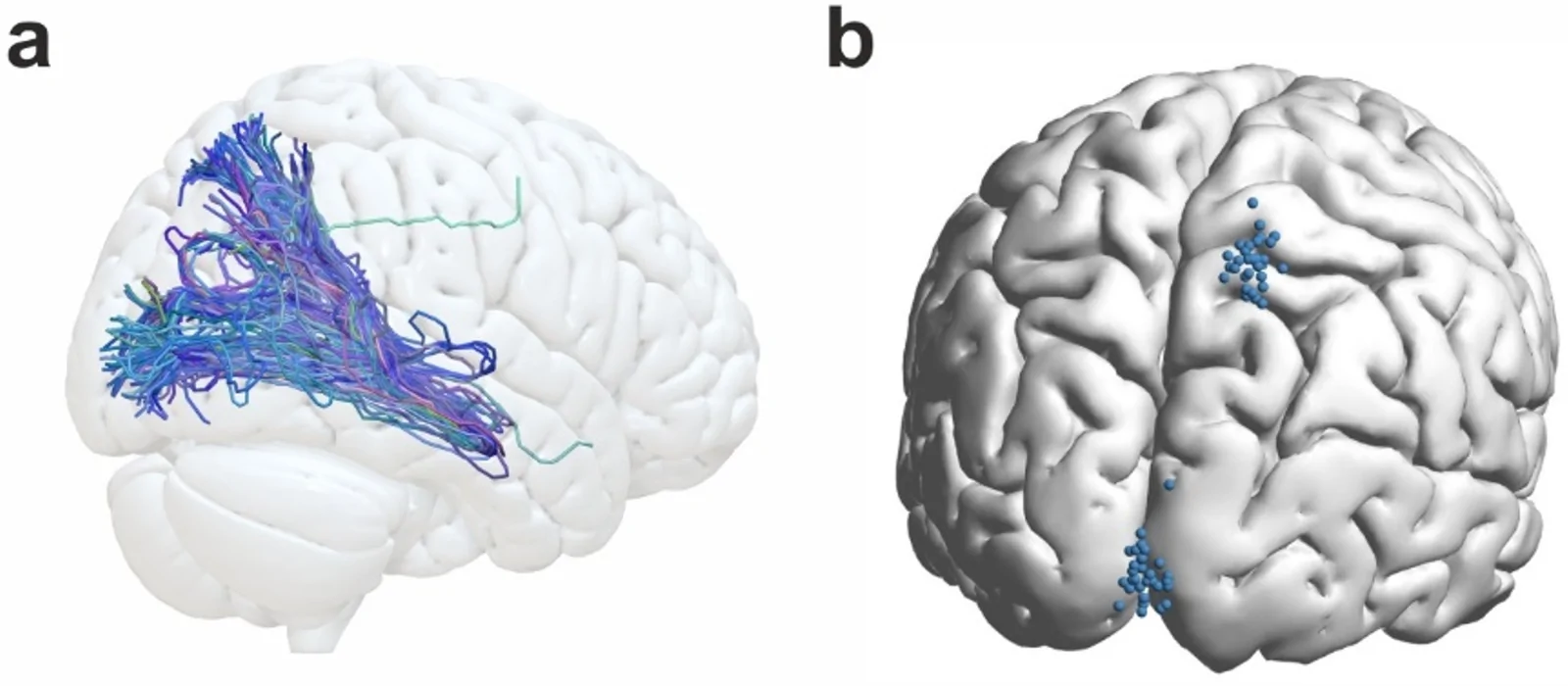
V1–IPS pathway reconstruction and TMS targets. (a) Reconstruction of the V1–IPS stream in a representative participant. (b) Tractography-based individualized cortical stimulation sites for each participant at the two TMS targets.

Individual V1–IPS ccPAS target points, derived from subject-specific white-matter tract reconstructions (Fig. 4a), are shown in Figure 4b. The mean rMT across subjects was 45.04% of MSO (± 6.67%). A one-way repeated measures ANOVA comparing rMT intensities across the three ccPAS sessions yielded a non-significant effect (F(2,62) = 2.76, p = 0.071, ηp² = 0.082).

Shapiro–Wilk test indicated that mean accuracy and RTs were normally distributed for both the global shape task (W = 0.952, p = 0.160; and W = 0.972, p = 0.548, respectively) and the local features task (W = 0.973, p = 0.580; and W = 0.951, p = 0.152, respectively).

### 3.3 H1.1: Effect of V1–IPS ccPAS on global shape discrimination performance

**Fig. 5.**
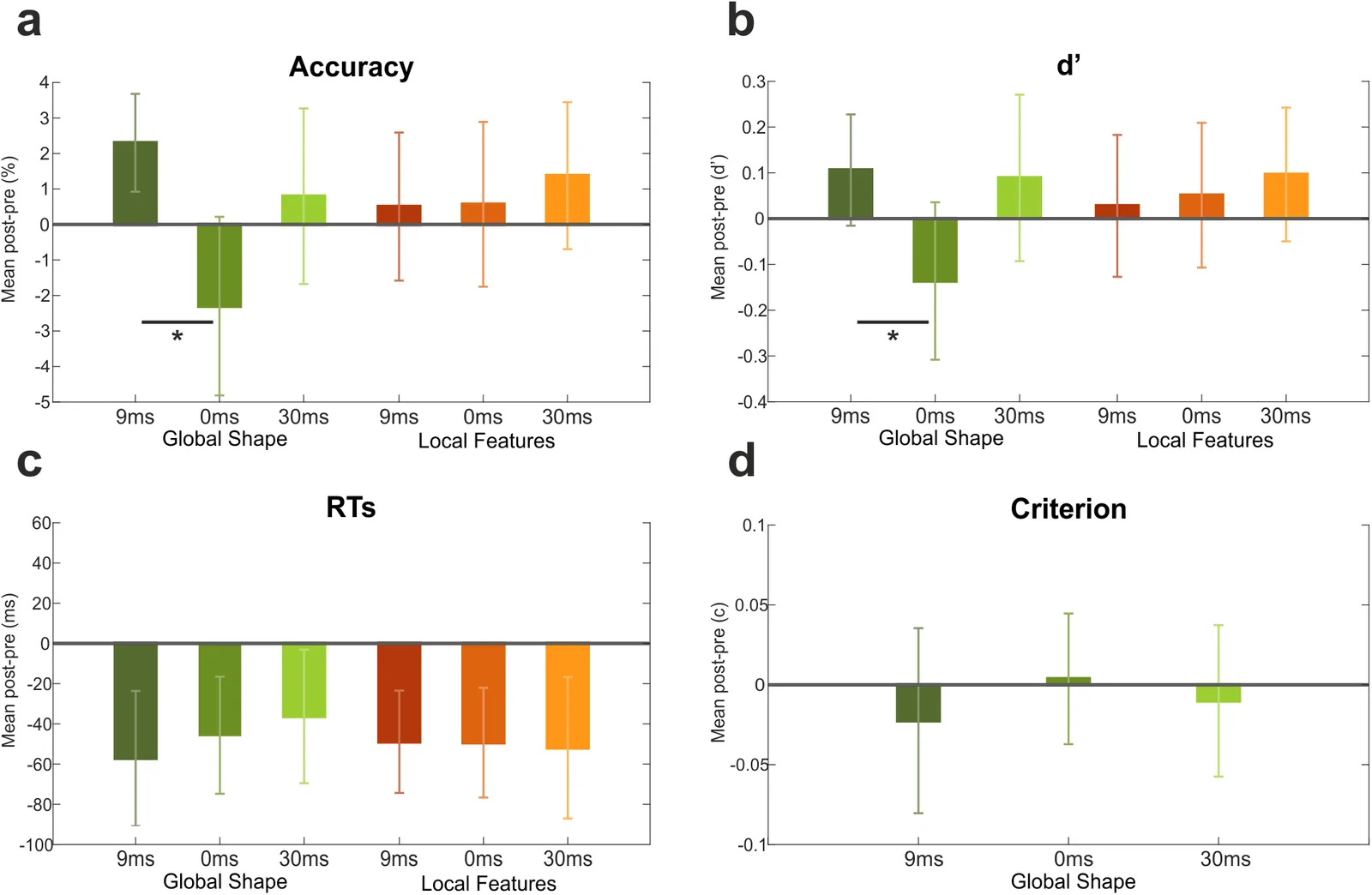
Preregistered analyses results. Behavioral results for accuracy (a), d’ (b), RTs (c), and criterion (d) are shown separately for the three ccPAS protocols and the two tasks. Bars display mean baseline-corrected (post–pre) values for each condition, with error bars indicating 95% confidence intervals. Green represents performance on critical trials in global shape tasks, whereas orange indicates performance in the local feature task. The asterisk marks the significant difference.

To address H1.1, we conducted a repeated measures ANOVA on baseline-corrected (post-ccPAS–pre-ccPAS) accuracy, RTs, and d’ values of Global shape trials, with “ccPAS condition” (V1–IPS 9 ms, V1–IPS 0 ms, V1–IPS 30 ms) and “Incongruency” (Totally vs. Partially incongruent trials) as within-subjects factors. Accuracy showed a significant main effect of “ccPAS condition” (F(2,62) = 4.966, p = 0.009, ηp² = 0.138). Post hoc comparisons indicated higher accuracy in the critical 9 ms ISI condition relative to the 0 ms control condition (t(31) = 3.503, p = 0.004, Cohen’s d = 0.619, mean 9 ms = 2.335, mean 0 ms = -2.330). No other comparison resulted significant (9 ms–30 ms: t(31) = 1.071, p = 0.877, Cohen’s d = 0.189, mean 30 ms = 0.790; 0 ms – 30 ms: t(31) = -1.815, p = 0.238, Cohen’s d = 0.322). The effect sizes were in the moderate range. Neither the main effect of “Incongruency” nor the “ccPAS condition” x “Incongruency” interaction was significant (F(1,31) = 2.866, p = 0.100, ηp² = 0.085; F(2,62) = 0.154, p = 0.858, ηp² = 0.005) (Fig. 5a and Supplementary Fig. S3a). Although all participants met the predefined inclusion criteria, visual inspection of the data suggested that one participant might represent a potential outlier. To assess the robustness of the finding, we, therefore, repeated the analysis after excluding this participant. The pattern of results remained unchanged, with the main effect of ccPAS condition and the post hoc difference between the 9 ms and 0 ms conditions remaining significant.

A similar pattern emerged for d’ scores, with a significant main effect of “ccPAS condition” (F(2,62) = 3.513, p = 0.036, ηp² = 0.102). Post hoc comparisons again showed higher d’ values in the critical 9 ms ISI condition compared with the 0 ms control condition (t(31) = 2.667, p = 0.036, Cohen’s d = 0.471, mean 9 ms = 0.127, mean 0 ms = -0.130). Other comparisons were not significant (9 ms–30 ms: t(31) = 0.314, p = 1, Cohen’s d = 0.055, mean 30 ms = 0.096; 0 ms–30 ms: t(31) = -1.874, p = 0.211, Cohen’s d = 0.331). No significant main effect of “Incongruency” or interaction was observed (F(1,31) = 3.769, p = 0.061, ηp² = 0.108; F(2,62) = 0.483, p = 0.619, ηp² = 0.015) (Fig. 5b and Supplementary Fig. S3b).

Analysis on RTs revealed no significant effects of “ccPAS condition,” “Incongruency,” or their interaction (“ccPAS condition”: F(2,62) = 0.516, p = 0.516, partial eta square = 0.016; “Incongruency”: F(1,31) = 0.452, p = 0.506, partial eta square = 0.014; “ccPAS condition” x “Incongruency” interaction: F(2,62) = 0.028, p = 0.972, partial eta square = 0.001) (Fig. 5c; and see Supplementary Fig. S3b, Supplementary Fig. S4, and Supplementary Fig. S5).

### 3.4 H1.2: Effect of V1–IPS ccPAS on response criterion in the global shape task

To test H1.2, we conducted a repeated measures ANOVA on baseline-corrected (post-ccPAS–pre ccPAS) c values of Global shape trials with “ccPAS condition” (V1–IPS 9 ms, V1–IPS 0 ms, V1–IPS 30 ms) as within-subject factor. Results showed no significant effect (F(2,62) = 0.269, p = 0.765, ηp² = 0.009) (Fig. 5d and Supplementary Fig. S3d).

### 3.5 H1.3: Effects of V1–IPS ccPAS on global shape versus local feature discrimination

To address H1.3, repeated measures ANOVA on baseline-corrected (post-ccPAS–pre-ccPAS) accuracy, RTs, and d’ values was performed, with “ccPAS condition” (V1–IPS 9 ms, V1–IPS 0 ms, V1–IPS 30 ms), “Task” (global shape task vs. local features task), and “Incongruency” (Totally vs. Partially incongruent trials) as within-subjects factors. Greenhouse–Geisser corrections were applied where appropriate.

Analysis on accuracy showed no significant main effect of “ccPAS condition” (F(1.57,48.57) = 2.208, p = 0.131, ηp² = 0.066), “Task” (F(1,31) = 0.462, p = 0.502, ηp² = 0.015), “Incongruency” (F(1,31) = 2.979, p = 0.094, ηp² = 0.088), or their interactions (Task x Incongruency: F(1,31) = 0.386, p = 0.539, ηp² = 0.012; Task x ccPAS condition: F(2,62) = 2.765, p = 0.071, ηp² = 0.082; Incongruency x ccPAS condition: F(2,62) = 1.094, p = 0.341, ηp² = 0.034; Task x Incongruency x ccPAS condition: F(2,62) = 0.658, p = 0.522, ηp² = 0.021) (Fig. 5a and Supplementary Fig. S3a).

d’ results showed no significant main effect of “ccPAS condition” (F(1.59,49.30) = 2.000, p = 0.155, ηp² = 0.061), “Task” (F(1,31) = 0.366, p = 0.549, ηp² = 0.012), “Incongruency” (F(1,31) = 3.834, p = 0.059, ηp² = 0.110), or their interactions (Task x Incongruency: F(1,31) = 0.712, p = 0.405, ηp² = 0.022; Task x ccPAS condition: F(2,62) = 1.349, p = 0.267, ηp² = 0.042; Incongruency x ccPAS condition: F(2,62) = 1.752, p = 0.182, ηp² = 0.053; Task x Incongruency x ccPAS condition: F(2,62) = 1.007, p = 0.371, ηp² = 0.031) (Fig. 5b and Supplementary Fig. S3b).

Analysis on RTs showed no significant main effects of “ccPAS condition” (F(2,62) = 0.149, p = 0.862, ηp² = 0.005), “Task” (F(1,31) = 0.102, p = 0.751, ηp² = 0.003), or “Incongruency” (F(1,31) = 0.791, p = 0.381, ηp² = 0.025). A significant “Task” × “Incongruency” interaction emerged (F(1,31) = 5.999, p = 0.020, ηp² = 0.162); however, post hoc comparisons did not reveal any significant difference (all p’s > 0.258). No other significant interactions emerged (Task x ccPAS condition: F(2,62) = 0.640, p = 0.531, ηp² = 0.020; Incongruency x ccPAS condition: F(2,62) = 0.320, p = 0.727, ηp² = 0.010; Task x Incongruency x ccPAS condition: F(2,62) = 0.351, p = 0.705, ηp² = 0.011) (Fig. 5c; and see Supplementary Fig. S3c, Supplementary Fig. S4 and Supplementary Fig. S5).

### 3.6 Exploratory analyses

To further investigate our results, we conducted additional exploratory analyses.

#### 3.6.1 Exploratory 1: Pre-ccPAS performance across conditions

To assess whether baseline performance differed across the three ccPAS sessions, we compared pre-ccPAS performance across conditions for the main dependent variables and tasks. Repeated measures ANOVAs were conducted separately for accuracy, d’, and RTs, with ccPAS condition (V1–IPS 9 ms ISI, V1–IPS 0 ms ISI, V1–IPS 30 ms ISI) and Task (global shape task, local features task) as within-subject factors. No significant main effects or interactions were found for any of the dependent variables. For accuracy, there was no significant effect of ccPAS condition (F(2,62) = 0.087, p = 0.916, ηp² = 0.003), no significant effect of Task (F(1,31) = 0.256, p = 0.616, ηp² = 0.008), and no significant ccPAS condition x Task interaction (F(2,62) = 2.964, p = 0.059, ηp² = 0.087). Similarly, for d’, there was no significant effect of ccPAS condition (F(2,62) = 0.047, p = 0.954, ηp² = 0.002), no significant effect of Task (F(1,31) = 1.086, p = 0.305, ηp² = 0.034), and no significant ccPAS condition x Task interaction (F(2,62) = 1.620, p = 0.206, ηp² = 0.050). For RTs, there was no significant effect of ccPAS condition (F(2,62) = 0.745, p = 0.479, ηp² = 0.023), no significant effect of Task (F(1,31) = 0.124, p = 0.727, ηp² = 0.004), and no significant ccPAS condition x Task interaction (F(2,62) = 0.820, p = 0.445, ηp² = 0.026). These results indicate that pre-ccPAS performance was comparable across the three stimulation conditions for the main dependent variables. Importantly, the order of ccPAS conditions was counterbalanced across participants, reducing the likelihood that systematic practice or fatigue effects influenced baseline performance across sessions.

#### 3.6.2 Exploratory 2: Post hoc sensitivity analysis of the ccPAS x Task interaction

Although the study was adequately powered to detect the main effect of ccPAS condition based on prior estimates, it may have been underpowered to detect interaction effects in the full factorial design of H1.3 (ccPAS condition x Task x Incongruency). Interaction terms in repeated measures ANOVA typically require larger sample sizes due to smaller expected effect sizes and increased variability. Therefore, the absence of a significant “ccPAS condition” × “Task” interaction should be interpreted with caution. A sensitivity analysis conducted using G*Power indicated that, given the present sample size (n = 32) and design (3 x 2 within-subjects factors), the study was powered to detect effect sizes of f = 0.231 (ηp² = 0.051) or larger for the “ccPAS condition” x “Task” interaction, assuming alpha = 0.05 and power = 0.95 (sensitivity analysis was conducted focusing on the ccPAS condition x Task interaction as this effect was central to the present hypothesis). The observed effect sizes for the interaction differed across outcome measures, with a moderate effect for accuracy (ηp² = 0.082) and smaller effect for d’ (ηp² = 0.042).

#### 3.6.3 Exploratory 3: Effect of V1–IPS ccPAS on local features discrimination performance

Following the significant main effect of “ccPAS condition” observed for H1.1 and the trend toward a “Task” × “ccPAS condition” interaction in H1.3 (F(2,62) = 2.765, p = 0.071, ηp² = 0.082), we conducted additional analyses focusing on the local features task. Specifically, a repeated measures ANOVA was performed on baseline-corrected (post-ccPAS–pre-ccPAS) accuracy, RTs, and d’ values for local features task trials, with “ccPAS condition” (V1–IPS 9 ms, V1–IPS 0 ms, V1–IPS 30 ms) and “Incongruency” (Totally vs. Partially incongruent trials) as within-subject factors.

Accuracy results showed no significant main effect of “ccPAS condition” (F(2,62) = 0.177, p = 0.838, ηp² = 0.006), “Incongruency” (F(1,31) = 0.323, p = 0.574, ηp² = 0.010), or their interaction (F(2,62) = 1.507, p = 0.229, ηp² = 0.046) (Fig. 5a and Supplementary Fig. S3a). A similar pattern was observed when considering d’ results, with no significant main effect or interaction (Incongruency: F(1,31) = 0.364, p = 0.551, ηp² = 0.012; ccPAS condition: F(2,62) = 0.124, p = 0.884, ηp² = 0.004; Incongruency x ccPAS condition: F(2,62) = 2.343, p = 0.104, ηp² = 0.070) (Fig. 5b and Supplementary Fig. S3b). RT results showed a significant “Incongruency” main effect (F(2,62) = 4.451, p = 0.043, ηp² = 0.126). Post hoc comparisons indicated that RTs in Totally incongruent trials improved more than in Partially incongruent trials (t(31) = -2.110, p = 0.043, Cohen’s d = 0.373, mean Totally = -58.123, mean Partially = -41.822). No other significant main effect (ccPAS condition: F(2,62) = 0.014, p = 0.985, ηp² = 0.000) or interactions emerged (Incongruency x ccPAS condition: F(2,62) = 0.721, p = 0.490, ηp² = 0.023) (Fig. 5c and Supplementary Fig. S3c).

#### 3.6.4 Exploratory 4: Pre–post changes in discrimination performance following V1–IPS ccPAS

Given the significant effects on ccPAS condition (H1.1), we conducted additional exploratory analyses to directly test whether performance significantly changed from pre- to post-V1–IPS ccPAS within each condition. To do so, we compared post- with pre-stimulation accuracy and d’ scores (rather than post–pre difference scores) using a series of Bonferroni-corrected paired t-tests. Analyses were performed separately for each task (global shape and local features task) and ccPAS condition (V1–IPS 9 ms ISI, V1–IPS 0 ms ISI, V1–IPS 30 ms ISI).

**Fig. 6.**
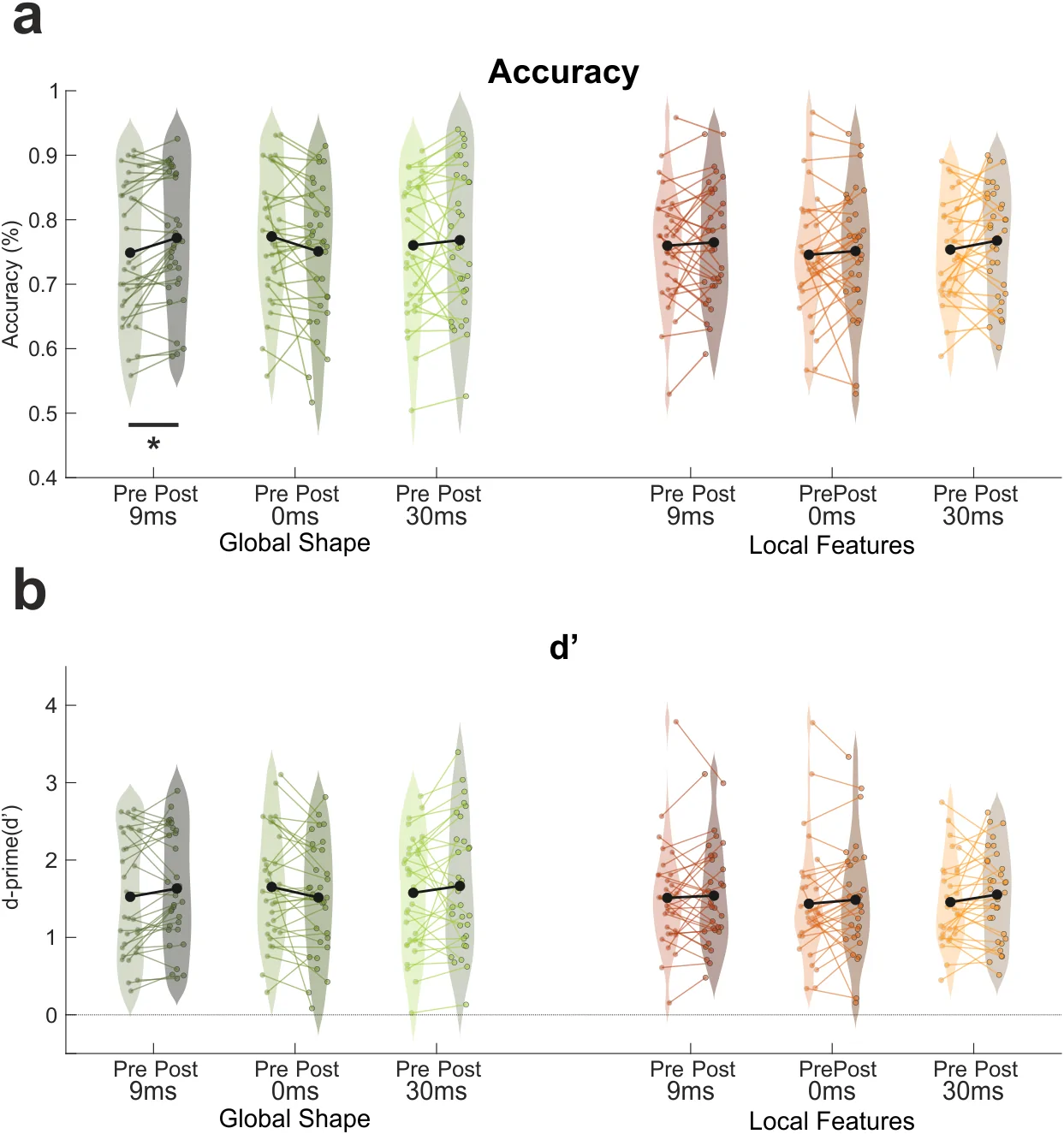
Exploratory analyses results (post- vs. pre-ccPAS). Raw behavioral results for accuracy (a) and d’ (b) values are presented separately for the three ccPAS protocols. Distribution and single-subject values are represented for each condition. Green represents performance on critical trials for the global shape task, while orange indicates performance on the local feature task. For each condition, both the distribution and individual subject values are displayed. Pre-stimulation distributions are shown in lighter shades, whereas post-stimulation distributions are shown in darker shades. Lines connect paired pre- and post-values for each participant. The asterisk marks the significant effect observed in the global shape task for the critical 9 ms ISI ccPAS condition.

Crucially, only the 9 ms ISI ccPAS condition yielded a significant post- vs. pre-stimulation increase in accuracy in the global shape task (t(31) = 3.274, p < 0.01, Cohen’s d = 0.579, mean post = 0.772, mean pre = 0.749), indicating that the performance improvement was selective to this condition (Fig. 6a). All other comparisons for accuracy (all p’s > 0.083), as well as all comparisons for d’ (all p’s > 0.097), did not show any statistically significant differences (Fig. 6b; see Supplementary Table S2 for a complete report of all statistical comparisons).

## 4 Discussion

The present study aimed to contribute to the ongoing debate on the role of the dorsal visual stream in global shape processing. To this end, we combined a causal manipulative approach (ccPAS) with individualized tractography (DTI) to strengthen V1-to-IPS dorsal stream connectivity.

Overall, the present results support the hypothesis that enhancing V1–IPS connectivity facilitates global shape discrimination. Specifically, the critical 9 ms ccPAS protocol led to improved performance on the global shape task, consistent with the view that the dorsal stream contributes directly to global shape representation (Ayzenberg & Behrmann, 2022a, 2022b; Ayzenberg & Behrmann, 2023; Ayzenberg et al., 2023; Bracci & Op de Beeck, 2016; Freud et al., 2016). More broadly, these findings challenge strictly ventral-only accounts of object shape representation, suggesting that dorsal stream mechanisms can also contribute to shape processing.

Results for H1.1 revealed a significant main effect of ccPAS condition on both performance measures (accuracy and d’) in the global shape task, indicating differential modulation across stimulation protocols, driven by greater improvement following the critical 9 ms ccPAS condition compared with the 0 ms control condition. Although this difference might, in principle, reflect changes in performance in the control condition, exploratory post- vs. pre- analyses showed that only the 9 ms ISI condition exhibited a significant improvement in performance, whereas the pre–post comparison for the 0 ms ISI condition was not significant. These exploratory findings are consistent with the interpretation that the clearest behavioral improvement occurred following the critical 9 ms ccPAS condition, although they should not be interpreted as proving definitive evidence that no change occurred in the control condition.

Importantly, the V1–IPS 0 ms ccPAS condition was meant as a control for non-specific TMS effects, and the absence of comparable changes in this condition supports the idea that the behavioral improvement observed after the 9 ms protocol cannot be attributed to non-specific factors.

Regarding temporal specificity, the findings provided only partial support for the time dependency of the critical 9 ms ccPAS condition. No significant difference emerged when comparing the effects of the 9 ms condition with those obtained using a 30 ms ISI on the same targets. However, this result should be interpreted in light of two considerations. First, the 30 ms ISI did not differ from the 0 ms control condition. Second, exploratory analyses revealed a significant post- vs. pre-improvement only for accuracy in the global shape task following the 9 ms ISI ccPAS condition, and no other significant differences across the other conditions or measures (accuracy, d’). Taken together, this pattern suggests that, although the direct comparison between the two active ISIs (9 ms vs. 30 ms) did not reach significance, the behavioral modulation was more consistent following the critical 9 ms protocol. The lack of a significant discrimination modulation after 30 ms ccPAS aligns with prior work showing that the 30 ms timing appears to be a plausible window for influencing backward IPS to V1 connections, yet not reliably changing perceptual accuracy, but instead affecting confidence-related measures (Di Luzio et al., 2022).

To further assess the genuineness of the effect found in the global shape discrimination, we examined its effect on response bias. This analysis provided an outcome-neutral test to determine whether the observed behavioral improvement could instead be explained by shifts in participants’ response criterion. Crucially, no significant main effect of ccPAS condition emerged, indicating that the improvement in performance cannot be attributed to changes in response criterion. This finding strengthens the interpretation that ccPAS modulated perceptual sensitivity rather than decision-related processes.

Given our hypothesis, the critical ccPAS protocol was expected to selectively modulate global shape rather than local feature processing. To test this prediction (H1.3), we compared its effects on global shape versus local features discrimination. This comparison also allowed us to address a possible attentional confound, given the well-established role of IPS in visual attention (Corbetta & Shulman, 2002). No significant ccPAS x task interaction emerged for either accuracy or d’. Although this finding does not provide formal statistical evidence for a dissociation between global and local processing, exploratory analyses were consistent with our hypothesis. Specifically, the V1–IPS 9 ms ccPAS protocol did not induce any significant change in local features discrimination. This was further supported by exploratory post- vs. pre-comparisons, which showed a significant improvement only in the global shape task following the 9 ms ISI ccPAS condition. Overall, this pattern is compatible with the possibility that strengthening the dorsal stream may have a stronger impact on global shape processing than on local feature processing, while making a generalized attentional account less likely.

The absence of a significant ccPAS x task interaction suggests that the observed effects should be interpreted with caution with respect to task specificity. One possible interpretation is that the dorsal stream may contribute more strongly to global shape recognition than to local feature analysis. In this view, the ccPAS-induced potentiation of this pathway may have preferentially enhanced processes underlying global configuration processing, consistent with previous evidence suggesting dorsal stream involvement in global shape recognition (Ayzenberg & Behrmann, 2022a, 2022b, 2023; Ayzenberg et al., 2023; Bracci & Op de Beeck, 2016; Freud et al., 2016).

Another possible explanation for the missing dissociation is that the present study lacked sufficient power to detect interaction effects of modest magnitude. Consistently, a sensitivity analysis indicated that the present design was powered to detect effects of ηp² ≈ 0.051 or larger for the ccPAS × task interaction. The observed effect sizes differed across measures, with ηp² = 0.082 for accuracy and ηp² = 0.042 for d′. While the effect for accuracy exceeded the minimum detectable threshold, the effect for d′ fell slightly below it, suggesting that the lack of a significant interaction may partly reflect limited sensitivity to smaller effects. Taken together, these results provide suggestive, but not definitive, evidence that V1–IPS ccPAS may have a stronger impact on global shape processing than on local feature processing. The consistency between the significant effect observed in the global shape task and the absence of effects in the local features task is consistent with a possible preferential influence of dorsal stream strengthening on global configuration processing. However, the lack of a statistically reliable interaction precludes a formal dissociation between processing levels, warranting cautious interpretation of task specificity.

Although our findings provide causal evidence that selectively strengthening V1–IPS connectivity modulates global shape processing, we do not suggest that this connection operates in isolation. Rather, the V1–IPS pathway likely represents one component of a broader distributed network supporting object shape processing. Consequently, one possible explanation for the relatively modest behavioral effect observed in the present study is that selectively targeting the V1 to IPS connection may not represent the most effective target to improve global shape discrimination. Other pathways, including direct inputs to IPS that bypass V1 and interactions between IPS and ventral visual areas, may also make substantial contributions to shape-related processing.

The absence of effects on reaction times, despite reliable improvements in accuracy and d′, suggests that ccPAS did not affect how quickly participants responded, but rather how effectively perceptual information was processed. In this view, strengthening V1–IPS connectivity may have enhanced the efficacy of feedforward signals conveying visual information to posterior parietal cortex, thereby improving the computation of spatial and relational properties that support global shape processing and object recognition (Ayzenberg & Behrmann, 2022a, 2022b). Consistent with this interpretation, strengthening the initial V1–IPS feedforward sweep may enhance the quality of the global structural representation computed in parietal cortex, in line with evidence that perceptual integration rapidly engages the dorsal pathway to guide subsequent visual processing (Liu et al., 2017). At the same time, because our ccPAS protocol targets two nodes within a highly recurrent network, the observed effect may in part reflect modulation of recurrent interactions between these regions, while remaining compatible with the temporal specificity of the 9 ms protocol. Because IPS has been proposed to compute object-centered part relations and to transmit this information to ventral visual areas for further categorization, enhanced input to this area may improve spatial relational computations critical for object discrimination, without necessarily altering the temporal dynamics of decision processes. Consistently, this interpretation aligns with drift–diffusion models, in which improved sensitivity is often associated with increased drift rate and minimal changes in reaction times (Georgiev et al., 2016; Ratcliff et al., 2015).

The present study offers new insights and contributions to existing literature. To the best of our knowledge, this is the first ccPAS study to investigate object processing. As such, it provides causal evidence on object shape processing in a field that has so far relied predominantly on correlational approaches. Beyond that, our protocol introduces an important methodological advance, namely the individualization of stimulation targets based on the reconstruction of each participant’s white-matter tracts. When the goal is to modulate a specific anatomical pathway, accounting for individual variability becomes critical. This is particularly relevant for relatively less characterized tracts, whose anatomical structure may differ substantially across individuals and thus could affect the outcome of the stimulation.

### 4.1 Limitations and future directions

The present findings should be interpreted in light of some limitations. First, as also highlighted by the exploratory analyses, the sample size is relatively small in relation to the complexity of the study design and the likely modest magnitude of the effect under investigation. Consequently, our findings may only partially capture the underlying mechanisms, particularly with respect to interaction effects. Second, the demanding nature of the experimental task resulted in a relatively high exclusion rate during the preliminary session, potentially resulting in a sample skewed toward participants proficient at this specific task, limiting the generalizability of the present findings to broader populations. Importantly, the task was not intended to retain only high-performing participants, but rather to exclude participants whose performance did not indicate reliable above-chance task engagement.

The task was specifically designed to probe fine-grained perceptual processes targeted by the ccPAS manipulation, but its high cognitive demands (including a substantial working memory component) likely contributed to the observed inter-individual variability in performance. Although these factors may have increased general task-related variability, they are unlikely to account for the specific pattern of ccPAS effects. Indeed, the selective improvements in accuracy and d’ in the absence of RT modulations, together with the lack of effects in control stimulation conditions, are more consistent with a genuine modulation of perceptual processing.

Further studies should, therefore, aim to consolidate and extend the present findings. Firstly, future work should rule out potential confounding effects arising from the task structure and task difficulty, seeking to replicate these findings using less demanding task variants that can be successfully completed by a wider range of participants. It would also be valuable to broaden the range of stimuli, for example, by including 2-dimensional non-graspable objects, to test whether the present effect holds beyond the specific stimulus set adopted here. More broadly, future work might also address the interplay between the ventral and dorsal streams in object processing. One possible target would be consolidating and exploring the role of IPS-LO (lateral occipital cortex), which has been proven to be critical for shape processing (Ayzenberg et al., 2023). Interestingly, in these studies, they found that activity in PPC predicted the one in the ventral pathway during a task of object recognition.

## 5 Conclusion

The present findings advance the ongoing debate on the neural basis of shape processing by providing causal evidence that the dorsal V1–IPS pathway contributes to global shape discrimination. Specifically, strengthening this pathway improved performance on a global shape task, supporting the view that dorsal stream can make a direct contribution to shape computations relevant for object recognition. At the same time, these results should not be taken to imply that shape processing is independent of the ventral stream, nor that dorsal mechanisms are sufficient on their own. Rather, our findings indicate that the dorsal stream plays a causal role in global shape processing, while the extent and nature of its interaction with ventral visual regions remain important questions for future research.

## Supplementary Material

Supplementary Material

## Data Availability

All raw and processed anonymized experimental data, as well as raw and preprocessed pilot data generated in the present study, together with the study materials, are publicly available at the following link: https://doi.org/10.17605/OSF.IO/84CAQ. All analysis code used in the present study is publicly available at: https://doi.org/10.17605/OSF.IO/84CAQ

## Footnotes

1 **Note. **Due to technical issues related to coil overheating, the original plan to deliver 180 paired pulses at 0.2 Hz was modified to use a lower stimulation frequency (i.e. 90 paired pulses at 0.1 Hz). The protocol remains effective and is consistent with previously published ccPAS protocols, including studies in the visual domain and beyond (Bevilacqua et al., 2023; Borgomaneri et al., 2023; Chiappini et al., 2022; Fiori et al., 2018; Turrini et al., 2023). Importantly, this change was implemented and approved by the recommender prior to data collection.
