# Supplementary material for: Causal evidence for dorsal stream involvement in object shape recognition: A tractography-guided ccPAS Registered Report study
Source: Imaging Neurosci (Camb). 2026 Sep 8;4:IMAG.a.1345. doi: 10.1162/IMAG.a.1345 (PMC13556797; doi:10.1162/IMAG.a.1345)
Supplement: Supplementary file 1 [file IMAG.a.1345_supp.pdf]

Supplementary information

Pilot data

Behavioral pilot

The behavioral task (see the "Behavioral task" section) has been pilot-tested with a group of participants in order to refine and validate its capability to assess comparable performance between global shape and feature trials and to avoid ceiling or floor effects. A total of twenty-seven participants (26 right-handed, 1 corrected left-handed; 15 females and 12 males; mean age ± standard deviation 24.5 years ± 4.9) were tested, all reporting normal or corrected-to-normal visual acuity and no history of neurological or psychiatric disorders. One participant was not included because of technical problems that prevented the completion of the task. According to the exclusion criteria (see the "Exclusion criteria" section), 12 participants were excluded from the sample because showing an accuracy below chance according to the one-tailed binomial test (59.38% accuracy, n=96, p= 0.041). 14 participants (13 right-handed, 1 correct left-handed; 5 females and 9 males; mean age ± standard deviation 24.8 years ± 5.7) were then included in the final sample of the pilot analysis. According to the Shapiro-Wilk test, the distribution of both mean RTs and accuracy showed a normal distribution ($W=0.929$, $p=0.295$; $W=0.919$, $p=0.21$). To validate our task prior to implementing the analysis plan, we conducted some statistical analysis on behavioral measures. Accuracy, RTs, and d' values were analyzed using three 2x2 repeated measures ANOVAs with Task ("Global shape task" trials; "Local features task" trials) and Incongruency of the items-to-match with the sample (totally incongruent; partially incongruent) as within-subjects factors. No significant main effects were found in both analyses. Results showed a significant Task x Incongruency interaction for accuracy ($F(1,13)=16.744$, $p=0.001$, $\eta p2=0.563$). Bonferroni-corrected post hoc analysis showed that the performance in the "Global shape task" did not differ between totally and partially incongruent trials, whereas in the "Local features task" emerged a significant difference ($p=0.002$; see supplementary Fig. S1a). ANOVA on d' values showed a significant main effect of "Incongruency" ($F(1,13)=15.51$, $p=0.002$, $\eta p2=0.544$) and a significant Task x Incongruency interaction ($F(1,13)=5.08$, $p=0.042$, $\eta p2=0.281$), where Bonferroni-corrected post hoc comparisons showed a significant difference between totally and partially incongruent trials for the "Local features task" only ($p<0.001$; see supplementary Fig. S1c). These findings suggest that the spatial configuration (i.e., global shape) is processed independently of the local features in "Global shape task". Finally, a two-tailed paired-samples t-test on c values comparing the two tasks attested no significant difference ($t(13)=0.014$, $p=0.989$, Cohen's $d=0.003$; see supplementary Fig. S1d). To conclude, our pilot data show that we can reliably measure the behavioral performance concerning global shape and local feature tasks, that the performance is comparable, and no ceiling or floor effects occurred.

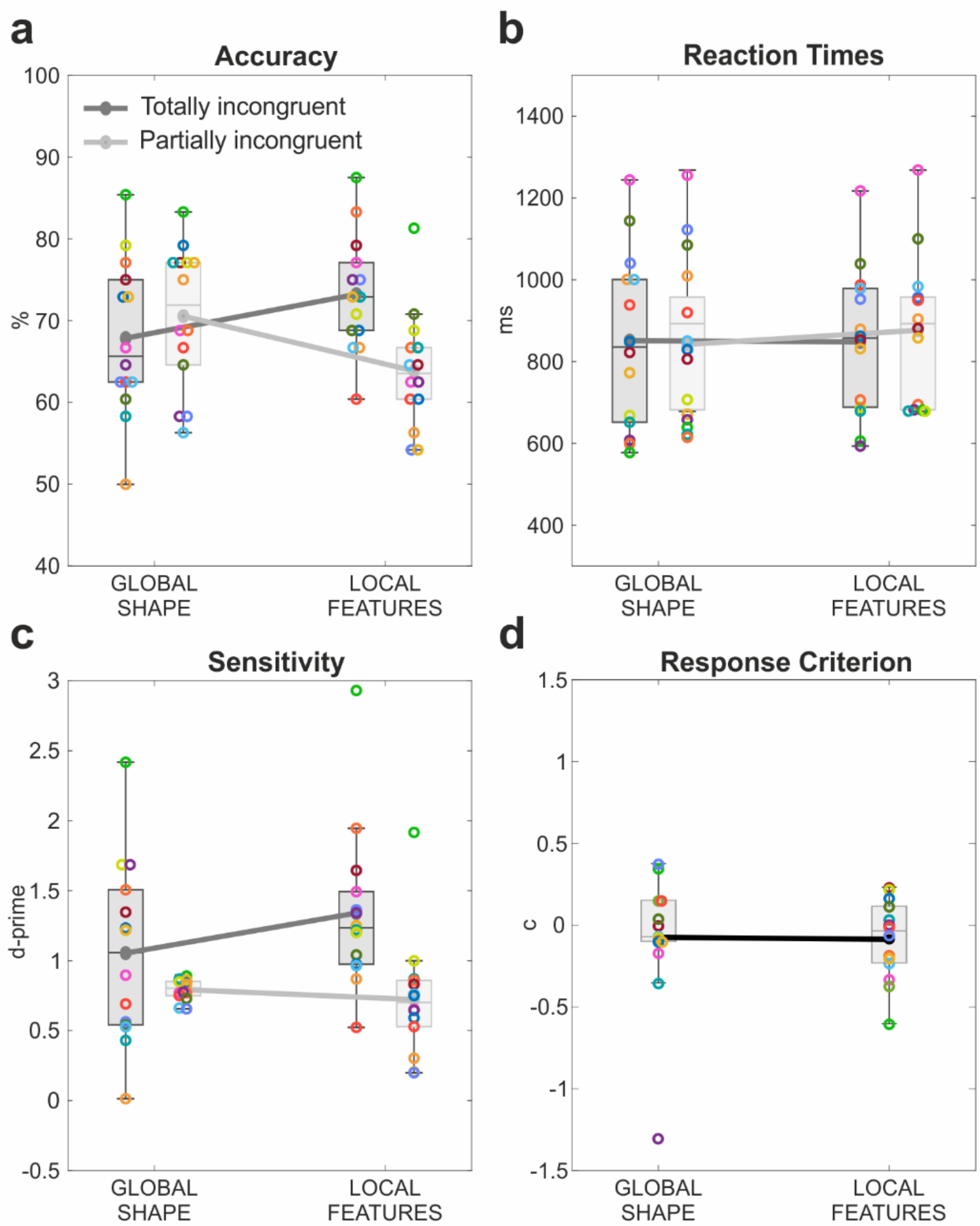

Supplementary Figure S1 | Behavioral pilot results (n=14) Graphical representation of results divided for "Global shape task" trials and "Local features task" trials reporting: **a.** Accuracy; **b.** Reaction Times; **c.** d' values; **d.** c values. Colored dots represent single-subject data, gray dots represent the mean value for each condition.

DWI pilot

The MRI acquisition protocol and the MRI preprocessing pipeline have been pilot-tested with one participant in order to verify the parameters of anatomical images acquisition, to shape the pipeline based on acquired signal, and to validate the feasibility of the whole protocol. The MRI acquisition protocol succeeded in acquiring a 3D T1-weighted (T1w) Turbo-field echo image and diffusion-weighted (DWI) data. Moreover, the pipeline analysis, with all the standardized pre-processing steps described in the "Materials and Methods'' section, was successfully applied. The employed IPS ROI was correctly contained within the selected atlas regions. The reconstruction of the tract of interest in the subject space was successfully performed in TrackVis (as displayed in supplementary Fig. S2).

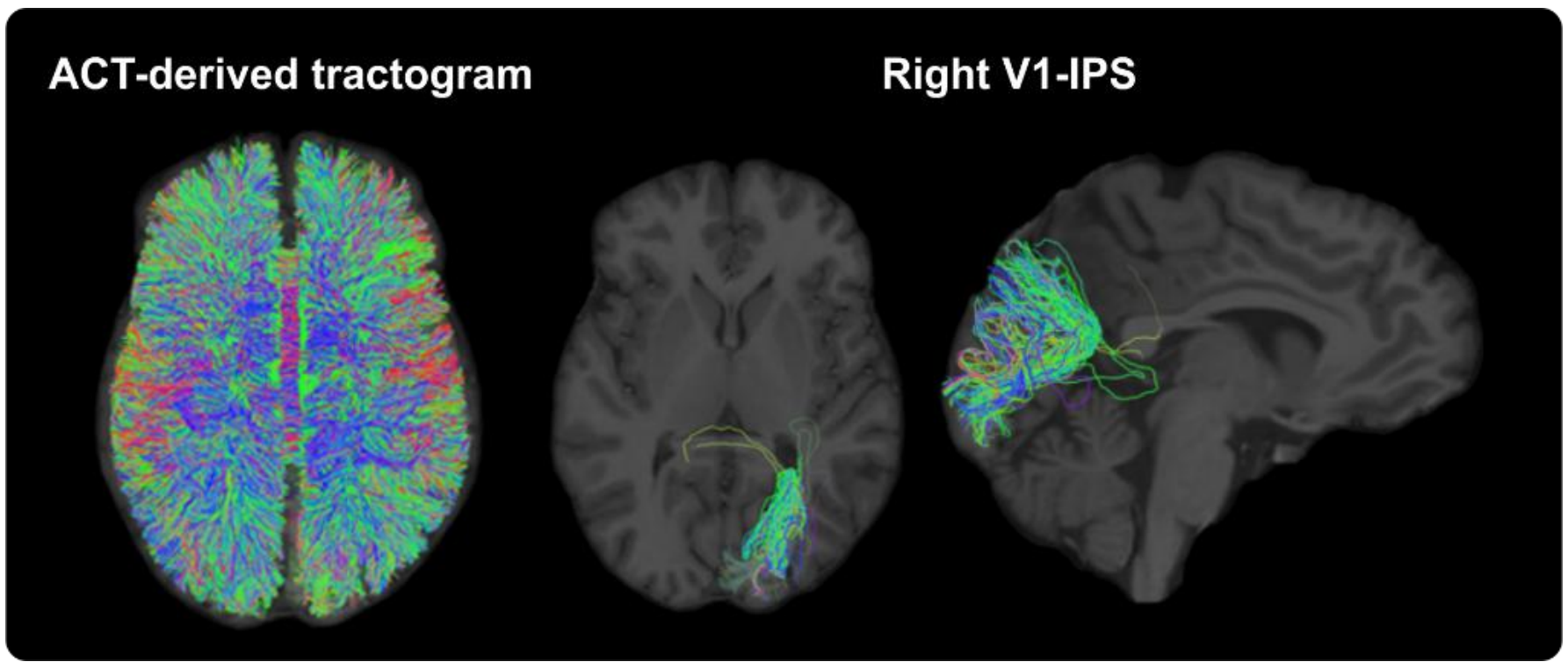

Supplementary Figure S2 | DWI pilot results (n=1). Visual representation of anatomical dissection of WM bundle of interest for tractography-informed neuronavigation in a single subject.

Additional results

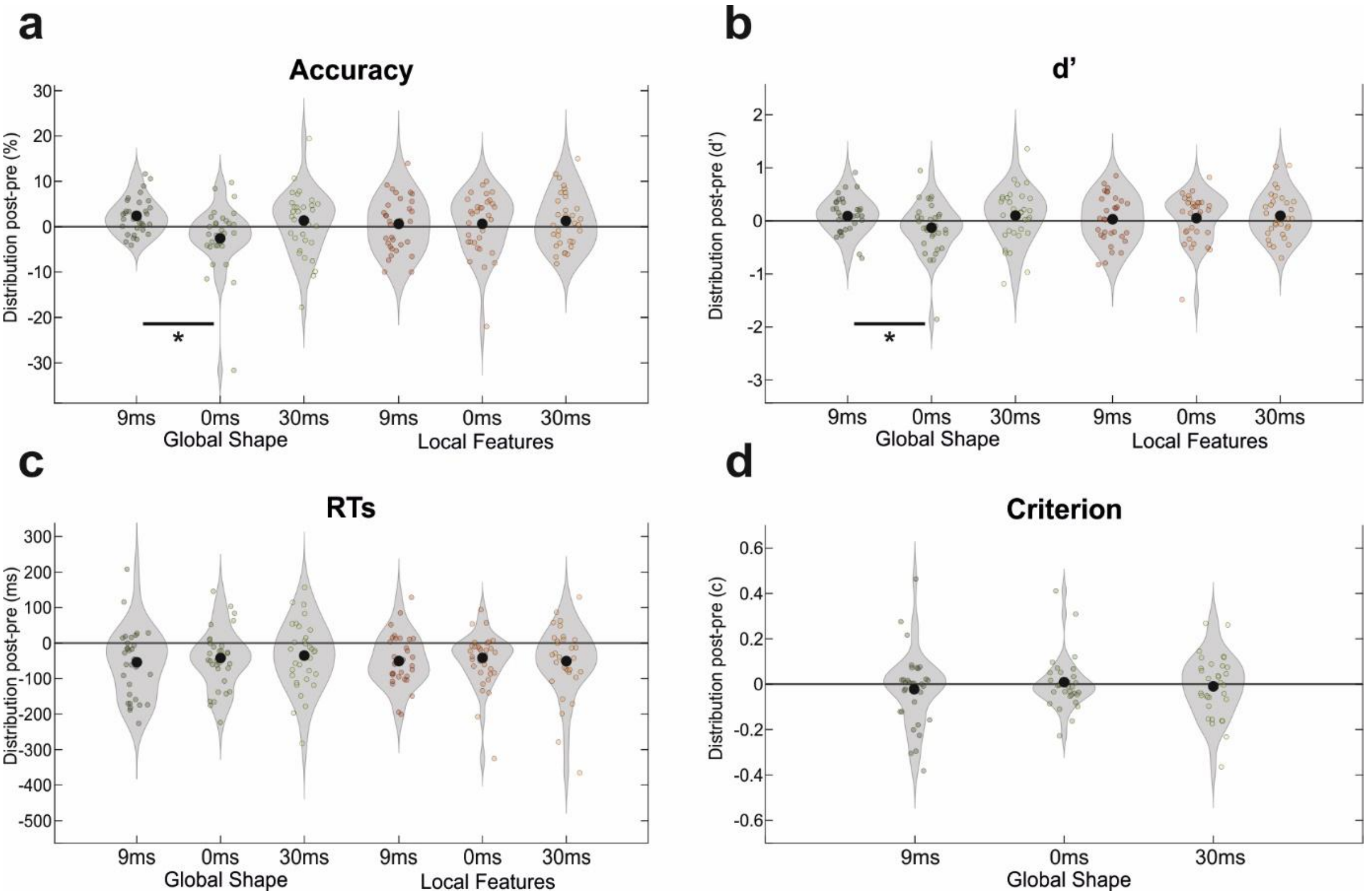

Supplementary Figure S3 | Preregistered analyses results: data distributions. Behavioral results for accuracy (**a**), d' (**b**), RTs (**c**), and criterion (**d**) are shown separately for the three ccPAS protocols and the two tasks. Violin plots with overlaid dots depict the distribution of individual participant baseline-corrected (post-pre) values, with the width of the violins reflecting the relative density of observations. Green represents performance on critical trials in global shape tasks, whereas orange indicates performance in the local feature task. Black dots represent the mean value of each distribution

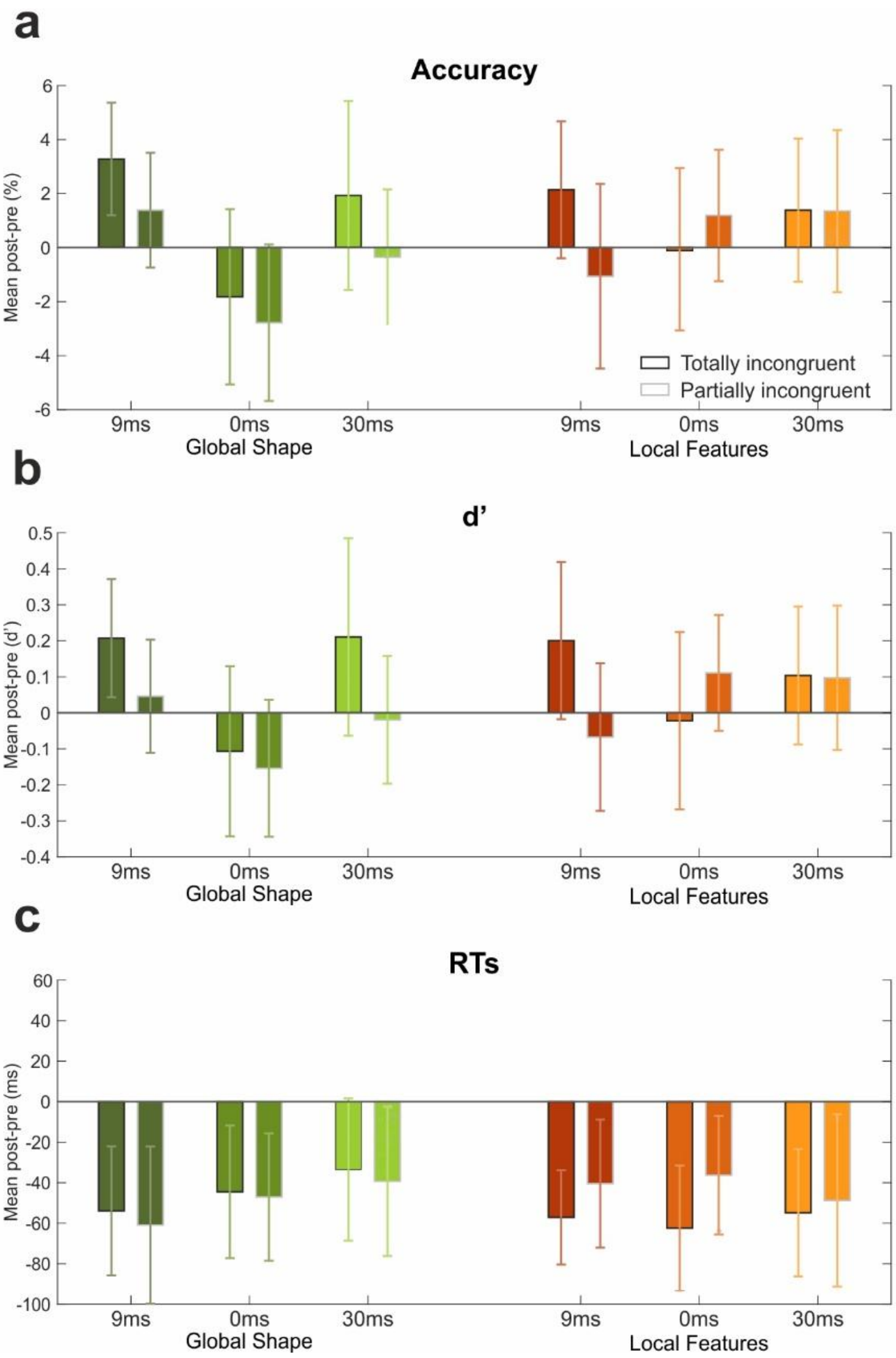

Supplementary Figure S4 | Additional planned analyses results. Behavioral results for accuracy (**a**), d' (**b**), and RTs (**c**) are shown separately for the three ccPAS protocols, the two tasks, and the two trial types. Bars display mean baseline-corrected (post-pre) values for each condition, with error bars indicating 95% confidence intervals Green represents performance on critical trials in global shape task, whereas orange indicates performance in the local feature task. Totally incongruent trial values are shown with light gray contours, and partially incongruent trial values with dark gray contours.

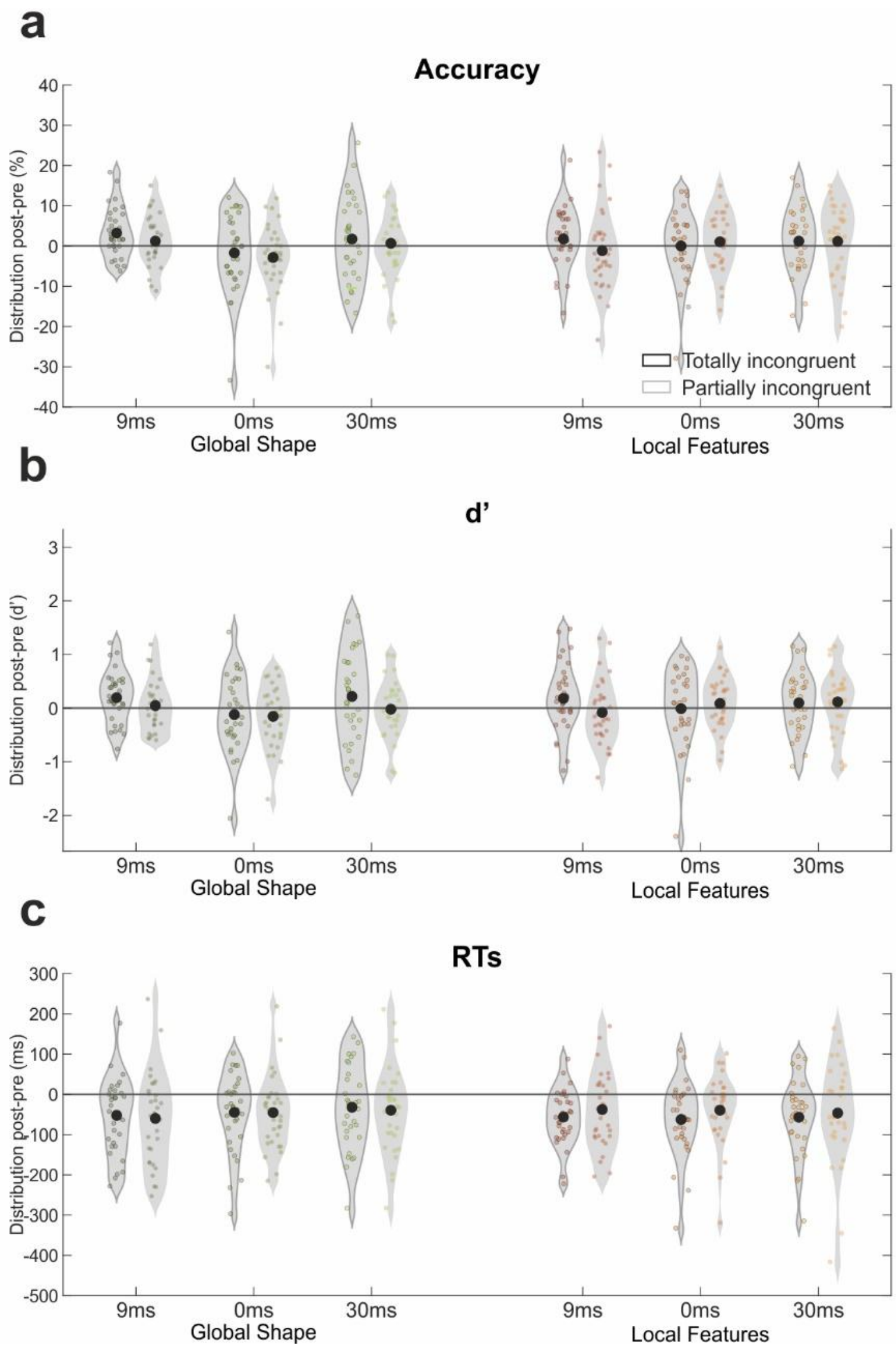

Supplementary Figure S5 | Additional planned analyses results: data distributions. Behavioral results for accuracy (**a**), d' (**b**), and RTs (**c**) are shown separately for the three ccPAS protocols, the two tasks, and the two trial types. Violin plots with overlaid dots depict the distribution of individual participant values. Green represents performance on critical trials in global shape task, whereas orange indicates performance in the local feature task. Totally incongruent trial values are shown with light gray contours, and partially incongruent trial values with dark gray contours. Black dots represent the mean value of each distribution

Supplementary Table S1: **Participant characteristics and experimental parameters**

Subj.ID	Age	Sex	Educ.	LQ	ccPAS protocol			Hand	Stimulation Coordinates (MNI)						rMT (%MSO)		
					sess.I	sess.II	sess.III		V1 x	V1 y	V1 z	IPS x	IPS y	IPS z	I	II	III
001	22	F	16	87.5	0	30	9	sx	25.2	-77.1	62.9	16.3	-108.8	6.0	35	42	43
002	19	F	15	100	30	9	0	sx	16.3	-108.8	6	25.2	-77.1	62.9	34	36	38
003	28	F	18	95.0	30	0	9	dx	12.7	-108.5	5.4	23.4	-72.1	68.7	44	45	43
004	21	F	16	61.3	0	9	30	sx	9.7	-107.9	3.3	24.6	-71.8	67	49	48	44
005	20	F	15	100	30	9	0	dx	10.3	-108.3	-2.4	18.6	-76.8	65.5	46	42	46
006	28	F	18	90.0	9	30	0	sx	4.5	-106.8	-2.6	26.4	-73.5	63	40	41	43
007	27	F	18	100	0	9	30	dx	14.6	-108.1	-1.1	27.8	-78.1	61.2	43	45	42
008	26	F	18	88.6	9	0	30	dx	14.5	-108.8	10.1	18.1	-71.5	65.6	52	49	46
009	20	F	15	100	0	30	9	dx	16.4	-108.8	4.9	25.4	-70	67.9	45	46	46
010	20	F	15	88.2	9	30	0	dx	7.3	-107.7	11.5	22.7	-64.5	70.2	33	40	40
011	22	F	16	94.6	9	0	30	sx	16.4	-108.2	7	20.1	-61.8	77.1	46	47	48
012	22	F	17	88.9	30	0	9	sx	11	-108.5	12.8	22.8	-65.1	73.7	47	50	48
013	20	F	15	100	30	9	0	sx	8.8	-109	8.8	23.3	-68.4	71.2	47	55	51
014	23	F	16	87.9	9	30	0	sx	10.3	-108.3	10.5	24.7	-73.5	59.9	44	41	57
015	24	M	15	93.9	0	9	30	dx	10.2	-107.1	-1.1	18.2	-73.2	69.1	49	48	49
016	22	F	16	100	9	0	30	sx	8.4	-108.8	5.2	23.8	-68.2	66.3	40	39	44
017	21	F	15	100	30	9	0	dx	10	-107.8	7.9	20	-70.1	70.6	40	40	39
018	28	F	18	100	0	30	9	dx	6.1	-108.3	4	20.7	-73.4	68.4	58	67	59
019	23	F	16	100	9	30	0	dx	15.7	-107.9	-0.1	26.2	-74	65.7	43	47	44
020	34	F	18	54.3	30	0	9	dx	10.3	-106.6	0	23.2	-75.3	61	50	53	55
021	27	F	18	94.4	0	9	30	sx	7.6	-108.7	3	26.2	-67.8	71.2	51	49	50
022	22	M	15	100	30	0	9	sx	9.6	-107	15.8	23.5	-69.5	66.7	55	55	53
023	24	M	16	70.6	9	0	30	dx	12.5	-109.2	7.3	23.7	-75.1	66.6	46	46	45
024	32	M	18	72.4	0	30	9	sx	8.5	-109.1	7.4	23.6	-76.7	63.4	54	53	52
025	22	F	16	100	9	30	0	sx	10.2	-108.4	13.8	21.6	-71.5	70	35	37	35

026	21	F	14	94.6	30	9	0	dx	11.6	-108.6	5.2	22.6	-72.4	68.6	39	39	38
027	22	F	15	77.8	9	0	30	sx	6.8	-107.6	3.6	22.5	-73.8	65	44	51	46
028	21	F	15	100	0	9	30	sx	10.1	-107.9	10	22.2	-70.6	70.9	44	41	46
029	22	M	16	90.0	0	9	30	dx	11	-109.1	1.4	20.2	-74.5	67	29	33	34
030	22	M	15	85.0	30	0	9	dx	14.2	-108.9	4.6	24.1	-67.4	65	55	53	49
031	27	M	19	82.9	30	9	0	sx	8.9	-108.6	12.4	30.4	-71.9	67.3	39	43	43
032	21	M	15	89.2	30	0	9	sx	8.9	-109	5.5	24.1	-71.3	68.4	47	46	40
033	23	F	16	92.3	9	0	30	dx	7.6	-109.4	3.1	19.1	-76.3	67.7	36	37	38

Supplementary Table S1 reports participant number; age; sex; years of education; LQ: Laterality Quotient of the Edinburgh Inventory Score (>40 for right-handed participant); ccPAS condition order (9ms ISI, 0ms ISI, 30ms ISI) across three sessions; participant's finger positioned on the "5" key at the beginning of the behavioral task.; cortical coordinates for ccPAS stimulation at V1 and IPS sites; rMT assessed in the three different sessions of the protocol.

Supplementary Table S2: Statistical results

Preregistered analyses

H1.1: 3 × 2 repeated-measures ANOVA on Global Shape Task trials with ccPAS condition (9, 0, and 30 ms ISI) and Incongruency (Totally vs Partially incongruent trials) as within-subject factors

Accuracy

	Main effect “ccPAS condition”	$F(2,62)=4.966$	$p=0.010$*	$\eta p^2=0.138$
	Main effect “Incongruency”	$F(1,31)=2.866$	$p=0.100$	$\eta p^2=0.085$
	“ccPAS condition x Incongruency”	$F(2,62)=0.154$	$p=0.858$	$\eta p^2=0.005$
Post hoc tests	9ms ISI-0ms ISI	$t(31)=3.50$	$p=0.004$*	Cohen’s d=0.619
	9ms ISI- 30ms ISI	$t(31)=1.07$	$p=0.877$	Cohen’s d=0.189
	0ms ISI – 30ms ISI	$t(31)=-1.82$	$p=0.238$	Cohen’s d=0.322

d’

	Main effect “ccPAS condition”	$F(2,62)=3.513$	$p=0.036$*	$\eta p^2=0.102$
	Main effect “Incongruency”	$F(1,31)=3.769$	$p=0.061$	$\eta p^2=0.108$
	“ccPAS condition x Incongruency”	$F(2,62)=0.483$	$p=0.619$	$\eta p^2=0.015$
Post hoc tests	9ms ISI-0ms ISI	$t(31)=2.667$	$p=0.036$*	Cohen’s d=0.102
	9ms ISI- 30ms ISI	$t(31)=0.317$	$p=1$	Cohen’s d=0.055
	0ms ISI – 30ms ISI	$t(31)=--1.874$	$p=0.211$	Cohen’s d=0.331

RTs

	Main effect “ccPAS condition”	$F(2,62)=0.516$	$p=0.599$	$\eta p^2=0.016$
	Main effect “Incongruency”	$F(1,31)=0.452$	$p=0.506$	$\eta p^2=0.014$
	“ccPAS condition x Incongruency”	$F(2,62)=0.028$	$p=0.972$	$\eta p^2=0.001$

H1.3: 3 × 2 × 2 repeated-measures ANOVA with ccPAS condition (9, 0, and 30 ms ISI), Task (Global shape vs Local features), and Incongruency (Totally vs Partially incongruent trials) as within-subject factors.

Accuracy

	Main effect “ccPAS condition”	$F(1.57,48.57)=2.208$	$p=0.131$	$\eta p^2=0.066$
	Main effect “Incongruency”	$F(1,31)=2.979$	$p=0.094$	$\eta p^2=0.088$
	Main effect “Task”	$F(1,31)=0.462$	$p=0.502$	$\eta p^2=0.015$
	“Task x Incongruency”	$F(1,31)=0.386$	$p=0.539$	$\eta p^2=0.012$
	“Task x ccPAS condition”	$F(2,62)=2.765$	$p=0.071$	$\eta p^2=0.082$
	“Incongruency x ccPAS condition”	$F(2,62)=1.094$	$p=0.341$	$\eta p^2=0.034$
	“Task x Incongruency x ccPAS condition”	$F(2,62)=0.658$	$p=0.522$	$\eta p^2=0.021$

d'

	Main effect "ccPAS condition"	F(1.59,49.30)=2.000	p=0.155	ηp²=0.061
	Main effect "Incongruency"	F(1,31)=3.834	p=0.059	ηp²=0.110
	Main effect "Task"	F(1,31)=0.366	p=0.549	ηp²=0.012
	"Task x Incongruency"	F(1,31)=0.712	p=0.405	ηp²=0.022
	"Task x ccPAS condition"	F(2,62)=1.349	p=0.267	ηp²=0.042
	"Incongruency x ccPAS condition"	F(2,62)=1.752	p=0.182	ηp²=0.053
	"Task x Incongruency x ccPAS condition"	F(2,62)=1.007	p=0.371	ηp²=0.031

RTs

	Main effect "ccPAS condition"	F(2,62)=0.149	p=0.862	ηp²=0.005
	Main effect "Incongruency"	F(1,31)=0.791	p=0.381	ηp²=0.025
	Main effect "Task"	F(1,31)=0.102	p=0.751	ηp²=0.003
	"Task x Incongruency"	F(1,31)=5.999	p=0.020*	ηp²=0.162
	"Task x ccPAS condition"	F(2,62)=0.640	p=0.531	ηp²=0.020
	"Incongruency x ccPAS condition"	F(2,62)=0.320	p=0.727	ηp²=0.010
	"Task x Incongruency x ccPAS condition"	F(2,62)=0.351	p=0.705	ηp²=0.011
Post hoc tests	GS T-GS P	t(31)=0.673	p=1	Cohen's d=0.119
	GS T-LF T	t(31)=1.353	p=1	Cohen's d=0.239
	GS T- LF P	t(31)=-0.171	p=1	Cohen's d=0.030
	GS P- LF T	t(31)=0.738	p=1	Cohen's d=0.130
	GS P- LF P	t(31)=-0.578	p=1	Cohen's d=0.102
	LF T- LF P	t(31)=-2.110	p=0.258	Cohen's d=0.373

GS= Global Shape Task; LF= Local Features Task; T= Totally Incongruent Trials; P= Partially Incongruent Trials. Post hoc comparisons were Bonferroni-corrected.

Exploratory analyses

Exploratory 2: 3 × 2 repeated-measures ANOVA on Local Feature Task trials with ccPAS condition (9, 0, and 30 ms ISI) and Incongruency (Totally vs Partially incongruent trials) as within-subject factors

Accuracy

	Main effect “ccPAS condition”	F(2,62)=0.177	p=0.838	ηp²=0.006
	Main effect “Incongruency”	F(1,31)=0.323	p=0.574	ηp²=0.010
	“ccPAS condition x Incongruency”	F(2,62)=1.507	p=0.229	ηp²=0.046

d’

	Main effect “ccPAS condition”	F(2,62)=0.124	p=0.884	ηp²=0.004
	Main effect “Incongruency”	F(1,31)=0.364	p=0.551	ηp²=0.012
	“ccPAS condition x Incongruency”	F(2,62)=2.343	p=0.104	ηp²=0.070

RTs

	Main effect “ccPAS condition”	F(2,62)=0.015	p=0.985	ηp²=0.000
	Main effect “Incongruency”	F(1,31)=4.451	p=0.043*	ηp²=0.126
	“ccPAS condition x Incongruency”	F(2,62)=0.721	p=0.490	ηp²=0.023
Post hoc tests	Totally-Partially	t(31)=-2.110	p=0.043*	Cohen’s d=-0.373

Exploratory 3: T-test post vs. pre for each ccPAS condition

Accuracy

	GS 9ms post vs. pre	t(31)=-3.274	p=0.003*	Cohen’s d=0.579
	LF 9ms post vs. pre	t(31)=-0.475	p=0.638	Cohen’s d=0.084
	GS 0ms post vs. pre	t(31)=-1.792	p=0.083	Cohen’s d=-0.317
	LF 0ms post vs.pre	t(31)=-0.480	p=0.635	Cohen’s d=0.085
	GS 30ms post vs. pre	t(31)=-0.631	p=0.533	Cohen’s d=0.111
	LF 30ms post vs. pre	t(31)=-1.301	p=0.203	Cohen’s d=0.230

d’

	GS 9ms post-pre	t(31)=-1.711	p=0.097	Cohen’s d=0.302
	LF 9ms post-pre	t(31)=-0.353	p=0.727	Cohen’s d=0.062
	GS 0ms post-pre	t(31)=-1.553	p=0.131	Cohen’s d=-0.271
	LF 0ms post-pre	t(31)=-0.635	p=0.530	Cohen’s d=0.112
	GS 30ms post-pre	t(31)=-0.959	p=0.345	Cohen’s d=0.169
	LF 30ms post-pre	t(31)=-1.294	p=0.205	Cohen’s d=0.229

GS= Global Shape Task; LF= Local Features Task. Post hoc comparisons were Bonferroni-corrected.
